# Spatial heterogeneity and spatially varying determinants of childhood stunting in Northern Rwanda: A cross-sectional study to inform targeted interventions

**DOI:** 10.1371/journal.pone.0343772

**Published:** 2026-02-26

**Authors:** Clarisse Kagoyire, Albert Ndagijimana, Gilbert Nduwayezu, Jean Nepo Utumatwishima, Jean Pierre Mpatswenumugabo, Marie Anne Mukasafari, Diane Rinda, Vedaste Ndahindwa, Kristina Elfving, Gunilla Krantz, Torbjörn Lind, Ali Mansourian, Renée Båge, Ewa Wredle, Elias Nyandwi, Aline Umubyeyi, Jean Baptiste Ndahetuye, Petter Pilesjö

**Affiliations:** 1 Department of Earth and Environmental Sciences, GIS Centre, Lund University, Lund, Sweden; 2 Centre for Geographic Information Systems and Remote Sensing, College of Science and Technology, University of Rwanda, Kigali, Rwanda; 3 College of Medicine and Health Sciences, School of Public Health, University of Rwanda, Kigali, Rwanda; 4 Department of Clinical Sciences, Umeå University, Umeå, Sweden; 5 Department of Civil, Environmental and Geomatics Engineering, College of Science and Technology, University of Rwanda, Kigali, Rwanda; 6 Department of Public Health and Community Medicine, Institution of Medicine, Sahlgrenska Academy, University of Gothenburg, Gothenburg, Sweden; 7 Department of Clinical Sciences, Swedish University of Agricultural Sciences, Uppsala, Sweden; 8 Department of Veterinary Medicine, College of Agriculture, Animal Science and Veterinary Medicine, University of Rwanda, Nyagatare, Rwanda; 9 Department of Applied Animal Science and Welfare, Swedish University of Agricultural Sciences, Uppsala, Sweden; 10 Department of Pediatrics, Institution of Clinical Sciences, Sahlgrenska Academy, University of Gothenburg, Gothenburg, Sweden; Arizona State University, UNITED STATES OF AMERICA

## Abstract

Despite national progress, stunting remains prevalent in specific regions of Rwanda, highlighting the limitations of coarse-resolution data for effective mapping and intervention planning. This study explored optimal spatial resolution and analytical approach to capture localised dynamics and the multifactorial nature of stunting. A cross-sectional, population-based study was conducted in the Northern Province of Rwanda, focusing on children aged 1–36 months. Data were collected using structured questionnaires covering socio-demographic, economic, health, childcare, livestock factors and anthropometric measurements. Environmental characteristics were obtained from national datasets, while household geographic coordinates were captured using a customized mobile geodata platform (*emGeo*). After data cleaning, predictors were analysed using univariable and multivariable logistic regression as well as geographically weighted logistic regression (GWLR) to account for spatial heterogeneity. Among 601 children, stunting prevalence was 27% (boys 33.8%; girls 20.9%). GWLR improved model fit, increasing adjusted deviance explained from 34% to 39%. Significant predictors included child age (adjusted OR = 2.46; 95% CI: 1.78–3.39), male sex (OR = 2.83; 95% CI: 1.65–4.86), birthweight (OR = 0.71; 95% CI: 0.54–0.94), maternal autonomy (ability to refuse sexual intercourse; OR = 0.48; 95% CI: 0.27–0.86), inconsistent maternal social support (OR = 2.30; 95% CI: 1.20–4.42), household electricity access (OR = 0.48; 95% CI: 0.27–0.84) and handwashing facilities (OR = 0.21; 95% CI: 0.07–0.67). GWLR revealed substantial spatial heterogeneity in these factors, delineating areas where each factor matters most. This household-level, spatially explicit analysis reveals localised risk patterns often masked by aggregated national data. Prioritising context-specific interventions (such as electrification, hygiene promotion, and enhanced maternal social support), can enhance effectiveness. The proposed analytical workflow provides a model for addressing persistent stunting in other resource-limited settings.

## Introduction

Undernutrition remains a critical global health problem, particularly affecting children and mothers in low- and middle-income countries [1–3]. A common form of chronic undernutrition is stunting, defined as low height-for-age compared to a reference population, reflecting prolonged inadequate nutritional intake [1,2]. While global and African stunting rates have decreased overall, the absolute number of stunted children in sub-Saharan Africa has continued to rise, indicating persistent challenges related to nutrition, food security, and socioeconomic factors [4,5]. Childhood stunting has lasting consequences, including increased vulnerability to infections and chronic diseases, impaired cognitive and educational outcomes, reduced productivity, and sustained poverty across generations [2,6–8].Recent reports indicate notable progress in reducing maternal and child stunting in Rwanda, to some extent attributed to interventions such as *“The first 1000 days”* campaign and the *“Early Childhood Development (ECD)”* program [9]. However, the prevalence remains high, with about 33% of children under five years stunted countrywide, indicated by a height-for-age z-score (HAZ) below −2 SD (standard deviation) compared to the World Health Organization (WHO) reference population [10]. Therefore, despite national efforts, the challenge persists in identifying locally specific pattern of stunting severity and factors across communities and households [11,12], to plan for efficient intervention.

In addition to food deficit linked to poverty, stunting is associated with factors like inadequate diets, poor sanitation and hygiene, inadequate maternal and childcare practices, polluted water sources, limited healthcare access, low maternal education, and limited socio-economic opportunities [13–15]. Previous research on spatial determinants of stunting in sub-Saharan Africa, including Rwanda, has mainly relied on geographic data from Demographic and Health Surveys (DHS) [11,16–18]. The DHS data from Rwanda include geo-coordinates of 500 enumeration areas or clusters of households that span across the entire country. Each of these clusters contains approximately 26 surveyed households, out of several thousand households. To preserve respondent confidentiality, a random displacement of two and five kilometres is applied to urban and rural clusters, respectively. While DHS data hold valuable nation-wide information on population health, demography, and socioeconomic characteristics, this random displacement and the relatively small sample size can affect the spatial analysis at a smaller geographic scale [19].

Similarly, a study conducted in India using spatial clustering analysis identified district-level patterns of stunting coinciding with areas of high poverty, low female education, urbanization challenges, and low maternal body mass index, among other factors [20]. Uwiringiyimana et al. [11] used Bayesian geostatistical modelling to identify multiple socio-economic and demographic factors associated with spatial patterns of stunting among children under five years in Rwanda.

An interdisciplinary and spatially explicit approach using geographically weighted logistic regression (GWLR), a method that addresses spatial heterogeneity by enabling the analysis of localised risk factors, can help reveal determinants at fine-scale. GWLR extends traditional regression approaches by enabling coefficients to vary spatially, thus providing insights into local factors of stunting [21–23]. Moreover, there is a need to incorporate, in interdisciplinary manner, additional explanatory factors such as veterinary and animal sciences. The latter allows the exploration of direct and indirect nutritional effects related to animal-source foods, livestock management and zoonotic infections, potentially affecting child growth and development. Ownership of livestock such as cows or poultry may improve dietary intake and provide economic resources, potentially buffering against food insecurity [24]. To investigate these relationships, a cross-sectional survey was conducted.

This study, part of a broader research initiative, aimed to characterise the localised patterns of childhood stunting and examine a wide range of interdisciplinary risk factors. Specifically, it addressed two main objectives: (i) to develop and describe a survey methodology that fully integrates diverse determinants of stunting, including socio-demographic, health, environmental, livestock-related factors and household-level spatial data; and (ii) to evaluate the extent to which geographically weighted logistic regression (GWLR) enhances the understanding of spatially varying risk factors compared to traditional regression methods. The underlying hypotheses were that (a) childhood stunting is influenced by a complex, spatially heterogeneous set of factors that vary across communities, and (b) GWLR would improve model performance and reveal geographically distinct patterns of risk, thereby informing the design of more effective, context-specific interventions. Within this context, the study leverages household‑level geocoded data and GWLR to generate high‑resolution insights that can inform targeted interventions at district, sector, and community levels. The approach identifies geographically varying associations between stunting and modifiable determinants, including household electrification, hygiene infrastructure, and maternal empowerment, and demonstrates a reproducible workflow for advancing precision public health interventions in settings where data and resources are limited.

## Methods

### Study design and study area

**Fig 1:**
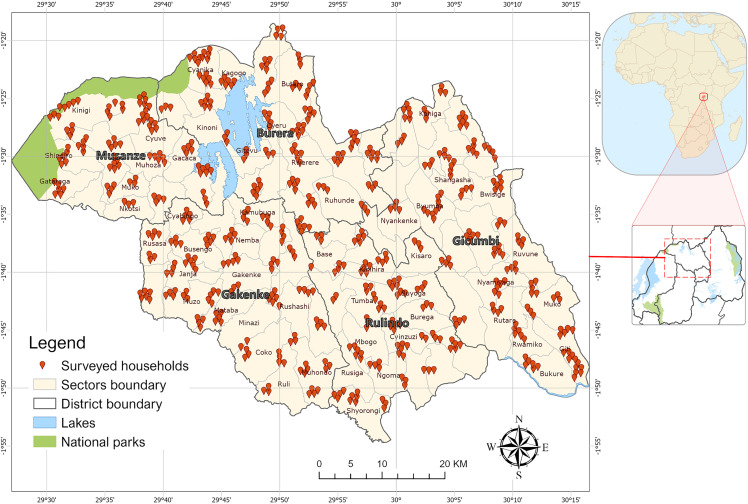
Study area in Northern Province, Rwanda. The Africa and Rwanda inset maps were created using the World Bank Official Boundaries dataset (CC BY 4.0).

A cross-sectional survey was conducted in 137 villages evenly distributed across the Northern Province of Rwanda, where stunting prevalence remains highest, with 40.5% of children below five years being stunted [10], collecting data on 612 independent variables. The selected study area is predominantly rural, with a population largely dependent on subsistence farming. The sampling design and location was decided after several stakeholders consultation meeting and field testing. The effective data collection was conducted from 24^th^ November to 30^th^ December 2021 across all five districts of the province: Burera, Gakenke, Gicumbi, Musanze, and Rulindo (Fig 1). The enumerators were organised into two teams: the first team collected individual and household-level data from all selected households, while the second team collected additional livestock and farming-related data from a subset of 160 households that owned dairy cows. The livestock data included livestock management with focus on feed, feeding practices and milk production, and veterinary public health aspects like biosecurity, zoonoses and food safety.

A web-based tool was developed, based on the internet-based information Management System for Environmental Protection and Disaster Risk Management (iMSEP) [25], an interoperable platform for geographical data collection, analysis, and sharing. The tool was integrated as an extension of *emGeo*, an android-based mobile GIS application within iMSEP, enabling geo-coded, real-time data collection and questionnaire administration [25]. The collected anonymised data were stored in a secure, access-controlled cloud-based environment that complied with all applicable data protection and ethical standards. This setup enabled timely sharing among authorized enumerators and facilitated quality control by researchers in Rwanda and Sweden. All data handling procedures, including storage, transfer, and access, were conducted in accordance with the Declaration of Helsinki and approved by relevant ethical review boards. Each enumerator used a GPS-enabled tablet with the questionnaire, developed from validated survey instruments. In addition to questionnaire data, geographic coordinates of all participating households were recorded.

### Community and demographic based sampling

The sample size was calculated based on an estimated stunting prevalence of 40.5% among children under five, with a 95% confidence interval (CI) and a margin of error of 5% (significance level, α = 0.05). The following formula was used:


$$n=\frac{Z^{\text{2}}\;\times \;p(\text{1}-p)}{\varepsilon^{\text{2}}}\;\times \text{DEFF}$$ (1)

where *n* is the sample size, *Z* the critical value for a 95% CI (1.96), *p* the estimated stunting prevalence (0.405), *ε* the margin of error (0.05), and *DEFF* is the design effect (1.5) to account for clustering. Considering an anticipated non-response rate (NR) of 10%, the adjusted final sample size was set to 615 households:


$$n_{adj}=n/(\text{1}-NR)=\text{5}\text{5}\text{3}.\text{1}\text{9}/\text{0}.\text{9}\approx \text{6}\text{1}\text{5}$$ (2)

A two-stage cluster random sampling strategy was used. The smallest administrative unit, the village, was used as the primary sampling unit. The study area is divided into 2744 villages. First, a grid map overlaying the study area was created to randomly select 137 villages (approximately 5% of all villages), ensuring even geographical distribution. Second, households within these villages were selected proportionally based on population density.

Within each village, households were selected using systematic random sampling. A sampling interval *(i)* was determined by dividing the total number of eligible households in the village by the required sample size from that village. The first household was randomly selected between the first household and the *i*^*th*^ household on the sampling frame obtained from community health workers. Subsequent households were selected systematically by adding the interval *i* to the previously selected household, until the required sample size was obtained.

### Study procedures and training

The study staff included six doctoral students, one post-doctoral fellow, and 12 enumerators. Eight enumerators (seven nurses and one medical doctor) supervised by four doctoral students and the post-doctoral fellow, collected household data on maternal and child health, nutrition, and biological samples. The remaining four enumerators (junior animal health scientists), working with two doctoral students in veterinary and agricultural sciences, collected livestock and farming-related data. All enumerators participated in training and received a standardised manual covering the study background, ethical considerations (including informed consent according to the Declaration of Helsinki), questionnaire administration using tablets, anthropometric measurements, and field quality assurance. Prior to starting the data collection, a pilot study was conducted to assess the feasibility of the data collection tools and logistics, leading to necessary adjustments.

#### Study participants’ enrolment.

The inclusion criteria were households with children aged between 1 and 36 months old. Households with a mother under the age of 18, or a mother who was unavailable or too sick to participate in the interview, were excluded from the study. When an eligible household was selected but the mother was temporarily unavailable, enumerators attempted revisits at different times of the day. If the interview still could not be completed, a replacement household was selected within the same village (the next nearest eligible household on the sampling list). This replacement protocol was pre-specified to preserve the planned sample size and spatial coverage while minimising selection bias. After written informed consent, each mother was interviewed using the digital questionnaire, and the child’s anthropometric measurements were taken.

#### Anthropometric measurements.

The anthropometric measurements were taken following standard procedures. The children’s height was measured using UNICEF height boards, and the weight was recorded using digital scales (SECA AG, Hamburg, Germany). The mid-upper arm circumference (MUAC) and head circumference were measured using standardised tape measures. For mothers, height was measured standing using measuring tapes, and weight was measured using digital scales (SECA AG, Hamburg, Germany).

### Outcome variables

The nutritional status of children below five years of age is usually assessed using anthropometric measurements [26]. Height-for-age z-score represents linear growth by comparing a child’s height to the average height of children of the same age in the reference population. Similarly, weight-for-height z-score (WHZ) represents body mass in relation to body length/height, while weight-for-age z-score (WAZ) focuses on body mass in relation to age, drawing a comparison between a child’s weight and the average weight of children of the same age in the reference population [26]. According to the WHO growth standards, a child with a HAZ score below −2 SD is considered stunted, indicating chronic malnutrition. A WHZ score below −2 SD represents wasting and indicates acute malnutrition, and a WAZ score below −2 SD suggests an underweight condition. This study collected data on all three indicators but focused primarily on stunting, as it is the most prevalent form of undernutrition in Rwanda, especially in the study area.

### Potential predictors of childhood stunting

A holistic number of potential predictors of childhood stunting, include data on household composition, socio-demographic and economic circumstances, child and maternal health and nutrition status, dietary intake, childcare practices, experiences of violence, dairy production and livestock management, as well as physical data like access to health care services. Altogether, 612 explanatory variables were collected and grouped into seven categories described briefly below.

#### Household socio-demographic factors.

Sampled household and socio-demographic factors included household location name, sex of household head, mother’s relationship with household head, religion, health insurance status, association membership, partner’s occupation and education, as well as water and sanitation indicators (e.g., drinking water source, water treatment practices, type of toilet, handwashing practices).

#### Household economic factors.

Economic factors included household income, assets, socio-economic classification locally known as *Ubudehe* [27], electricity access, ownership of transportation, housing quality (roof, walls, floor), household food insecurity measured by Household Food Insecurity Access Scale (HFIAS) [28], grown nutrient-rich crops, ownership of livestock, biosecurity measures on farms, and availability of animal-source foods like dairy products.

#### Child health and nutrition.

Collected variables included age, sex, birth weight, current illnesses such as diarrhoea, respiratory infections, chronic conditions, distance to health facilities, breastfeeding status, and types of food consumed. Dietary intake data were collected using the 24-hour dietary recall method [29], enabling the calculation of Minimum Dietary Diversity, an indicator of dietary quality and micronutrient adequacy as well as Minimum Acceptable Diet or Minimum Meal Frequency.

#### Childcare practices and presence of violence against children.

Variables that describe childcare practices and presence of violence against children included frequency and duration of periods when the child was left alone or with another child, identity of the person preparing and feeding the child, and child participation in nutrition programs or interventions such as vitamin A supplementation, deworming, micronutrient powder. Questions on violence against children included experiences of verbal or physical abuse, such as yelling, hitting, or name-calling [30].

#### Maternal health and presence of violence against mothers.

Maternal health indicators included maternal age, weight and height, marital status, number of miscarriages, antenatal care attendance, educational level, primary daily activities, and availability of social support. Data on exposure to physical, psychological, and sexual violence perpetrated by the husband or partner, and maternal mental health conditions (depression, anxiety, suicide risk) were also collected [31–33].

#### Milk production and animal husbandry factors.

Data collection covered milk production and cow husbandry factors, including feed resources, feeding practices, animal management practices, milk yield, milk production purposes, animal diseases, access to veterinary services, farm biosecurity, and milking and hygiene practices. Milk quality indicators (somatic cell counts, and antibiotic residues) were assessed to evaluate causes for low milk production and to identify the risk of exposure to unsafe milk, as milk is mostly used to supplement breastfeeding.

#### Environmental physical factors.

Physical factors represent the environmental characteristics and infrastructure linked to the geographical context of the area or community. These included climate (mean annual rainfall and temperature data from Rwanda Meteorological Agency, 1971–2017), elevation (extracted from a 10 × 10 m resolution digital elevation model), and proximity to infrastructure (major roads, health facilities, and markets). Distances to infrastructure were calculated using household GPS locations and geospatial datasets provided by the National Institute of Statistics of Rwanda (NISR). These environmental variables influence agricultural productivity, food availability, and accessibility to essential services.

### Data pre-processing

The collected data pre-processing phase included data cleaning, transformation, and discretization. Data cleaning involved removing duplicates, correcting typographical errors (such as administrative unit names), and treating outliers. Manual verification included reviewing photographs taken during data collection to confirm children’s vaccination based on the photographed vaccination cards and household construction materials, along with cross-checking anthropometric measurements using field notes. Logical imputation methods were applied in specific cases [34], such as inferring total years of schooling from educational levels or breastfeeding status from related responses.

Records with more than 60% missing data (n = 14, including eight records missing anthropometric data) were excluded, leaving 601 records. From the initial 612 predictor variables, only variables with less than 25% missing data were retained, resulting in 364 variables. Data scaling and normalization were subsequently performed prior to statistical analysis. The used administrative boundary polygons (sector- and district level) were obtained from the Rwanda MININFRA GeoPortal (accessed on 10 December 2024) [35,36].

### Data analysis

#### Exploratory data analysis.

Descriptive statistics were used to summarise the outcome and predictors, calculating central tendency (mean, median) and dispersion measures (range, interquartile range, standard deviation) for continuous variables, and frequency distributions for categorical variables. The prevalence of childhood stunting was first estimated, followed by statistical tests to identify significant relationships between predictors and stunting. For continuous variables, distributional assumptions were assessed using histograms and normality tests; Student’s t-tests were applied when approximate normality and homoscedasticity were met, whereas Wilcoxon rank-sum tests were used for skewed or non-normally distributed variables. Categorical variables were assessed using Pearson’s Chi-square or Fisher’s exact tests [37]. Variables with a *p*-value < 0.05 were considered statistically significant (S1-S8 Tables).

Given the complex interactions among multiple risk factors and their spatial variability, we adopted an interdisciplinary, multivariable analytical approach. To manage a large set of predictors (n = 364, after excluding missing data), and address multicollinearity inherent in spatial cross-sectional data, we implemented a two-stage variable reduction procedure. First, elastic net-regularised logistic with cross-validation was applied to select predictors with non-zero coefficients, leveraging its combined Lasso (L1) and Ridge (L2) penalties for feature selection and model stability [38–40]. In the second stage, a logistic regression model was fitted to the selected subset, followed by AIC-based backward elimination [41,42], iteratively removing variables until no further improvement in model fit was achieved. The resulting predictors formed the final feature set for subsequent univariable and multivariable logistic regression and GWLR analyses to evaluate associations with childhood stunting status. The dataset (N = 601) was randomly partitioned into training (80%; n = 480) and validation (20%; n = 121) subsets. Odds ratios (ORs) and 95% CIs were estimated for logistic regressions. The models performance was evaluated using standard classification metrics, including the area under the receiver operating characteristic curve (AUROC) and the area under the precision-recall curve (PR-AUC), which assess overall discrimination and performance in imbalanced data [43]. Sensitivity and specificity were calculated to measure the model’s ability to correctly identify stunted and non-stunted children, respectively.

As a sensitivity analysis for clustering under the village-based sampling design (the primary sampling unit), we fitted a mixed-effects logistic regression with a village-level random intercept, using the same fixed-effect predictors and coding as the final multivariable model. Fixed effects were summarised as adjusted odds ratios with 95% intervals (2.5^th^ –97.5^th^ percentiles), and the posterior standard deviation of the village-level random intercept was reported to quantify residual between-village heterogeneity.

#### Spatial statistical analysis.

Spatial and geostatistical analyses were performed using *Python 3.13.2*, enabling rigorous examination of childhood stunting factors at household and community levels. For visualisation, *ArcGIS Pro 3.2.2* was used to map spatial patterns by integrating georeferenced data on socio-demographic characteristics, economic indicators, and child and maternal health. Spatial autocorrelation measure, such as Moran’s I, Geary’s C, and Getis-Ord statistics [44–46], were used to determine whether households with similar characteristics (such as economic status or stunting prevalence) exhibited clustering or dispersion. Unlike traditional statistics, spatial analyses account for the interdependence among nearby observations [47]. For example, neighbouring households sharing a contaminated water source might exhibit similar nutrition outcomes, reflecting positive spatial autocorrelation, which refers to similarity among neighbouring observations [48,49].

Spatial heterogeneity is another key concept that describes systematic variation at fine scale in variables across geographic areas, commonly addressed using spatial regression models like spatial lag, spatial error, and geographically weighted regression (GWR) [21]. GWR extends ordinary least squares regression by allowing coefficients to vary locally [21,49]. For binary outcomes such as stunting (1 = stunted, 0 = not stunted), (GWLR) is appropriate [23,50]. The standard logistic regression model is:


$$\text{log}\left(\frac{p_{i}}{\text{1}-p_{i}}\right)=\beta_{\text{0}}+\;\sum_{j=\text{1}}^{m}\beta_{j}{\;X}_{ij}+\varepsilon_{i}$$ (3)

where *p*_*i*_ is the probability of stunting for an observation *i, β*_*0*_ is the intercept, *β*_*j*_ are the regression coefficients and *ε*_*i*_ is the error term. However, to account for spatial non-stationarity, GWLR modifies this model to allow coefficients to vary geographically, as follows:


$$\text{log}\left(\frac{p_{i}}{\text{1}-p_{i}}\right)=\beta_{\text{0}}\left(u_{i},\;v_{i}\right)+\;\sum_{j=\text{1}}^{m}\beta_{j}{\left(u_{i},\;v_{i}\right)\;X}_{ij}+\varepsilon_{i}$$ (4)

where *(u*_*i*_ *v*_*i*_*)* represents the geographical coordinates at location *i*, and *β*_*0*_ *(u*_*i*_ *v*_*i*_*)* and *β*_*j*_ *(u*_*i*_ *v*_*i*_*)* are the location-specific intercept and coefficients, respectively. Using 601 records and 26 selected predictors (plus the intercept), the GWLR model was calibrated using an adaptive bi-square kernel. The optimal bandwidth was selected through cross-validation to ensure an appropriate balance between bias and variance. The model diagnostics, including the effective number of parameters, log-likelihood, Akaike Information Criterion (AIC), and deviance explained, were used to evaluate model performance [51]. An adaptive and bi-square kernel allows the size (or bandwidth) of the kernel to adjust depending on the density of observations around a location, with the weights declining in a quadratic manner as one moves away from the location of the observation of interest [22]. Following model calibration, local parameter estimates from GWLR were spatially visualised to assess their geographic variation. Point-level outputs were interpolated to a 2 km × 2 km grid and aggregated to sector-level administrative units, enabling spatial comparison across the study area. The maps were generated using Python with GeoPandas, Matplotlib and Seaborn libraries.

The study was reported in accordance with the Strengthening the Reporting of Observational Studies in Epidemiology (STROBE) guidelines, and a completed checklist (Supplementary Material: S1 Checklist*)*.

### Ethical considerations

Ethical approval was obtained from the University of Rwanda College of Medicine and Health Sciences Institutional Review Board (reference No. 181/CHMS IRB/2021), as well as the Swedish Ethical Review Authority (reference No. 2021_04055). Authorization to conduct data collection was obtained from designated government institutions, including the National Institute of Statistics of Rwanda (reference No 0971/2020/10/NISR and No 0295/2021/10/NISR), the Ministry of Local Governance (reference No 0779/07.01), and the Ministry of Health (reference No 20/4184/DPMEHF/2021 and No NHRC/2020/PROT/047). Details on measures taken to protect privacy and confidentiality were communicated, before the participant signed the consent form to confirm their participation in the study. To ensure the privacy and confidentiality of the participants, the interview was conducted in a safe and private environment. Additionally, a data anonymization protocol was implemented to minimize the risk of participant identification. This anonymization process ensured that the data remained untraceable during both the data analysis and the publication of the study results. Study staff offered to take any mother or child who showed signs of acute malnutrition or severe anaemia as well as maternal major depressive disorder to a nearby health centre.

## Results

The four key results of the analyses are summary descriptive statistics on stunting prevalence across the five districts (S1-S8 Tables), statistical binary associations between stunting and individual predictors (S9 Table), multivariable logistic regression, evaluating the combined effect of predictors on stunting and spatial analysis using GWLR to explore geographical variations in stunting risk factors across the Northern Province.

### Stunting, wasting, and underweight: sex- and district-specific patterns

**Fig 2:**
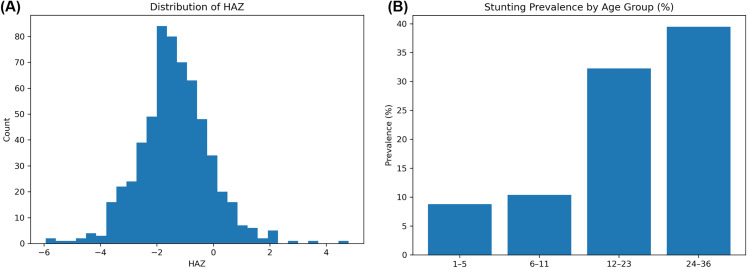
Childhood stunting patterns in the Northern Province of Rwanda. **(A)** Distribution of height-for-age z-scores (HAZ) showing an approximately normal distribution, with a substantial proportion of children below −2 standard deviations from the WHO growth standard, indicating stunting prevalence. **(B)** Prevalence of stunting by age group (in months).

**Fig 3:**
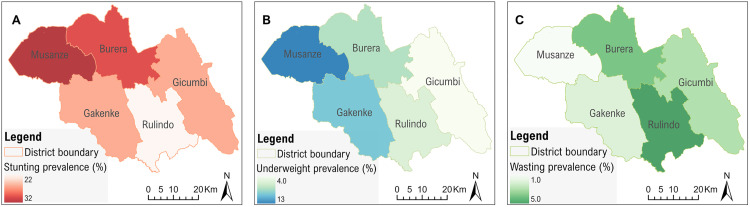
Spatial distribution of undernutrition indicators across the study area. (A) stunting (% of children with height-for-age below −2 SD), (B) underweight (% of children with weight-for-age below −2 SD), and (C) wasting (% of children with weight-for-height below −2 SD). Maps created using ArcGIS Pro 3.2.2 using study data and administrative boundary polygons (Rwanda MININFRA GeoPortal; sector- and district-level boundaries).

Among 601 children with complete data, 27% (95% CI: 23.7–30.8) were stunted (Fig 2A). The prevalence of stunting across age groups (in months) showed a marked increase with age, ranging from below 10% among children aged 1–6 months to approximately 40% among those aged 25–36 months (Fig 2B). Overall, stunting was more prevalent in boys (33.8%) than girls (20.9%). Musanze district exhibited the highest prevalence (approximately 33%), whereas Rulindo district had the lowest rate (Fig 3A). Only 3% of all children were found to be wasted (WHZ <−2 SD), while 7% were underweight (WAZ <−2 SD) (Fig 3B and 3C). The co-occurrence of stunting and wasting was low, with 6 children out of 601 being both stunted and wasted, while 32 children (5.3%) were both stunted and underweight.

### Descriptive and univariable logistic analysis of stunting

#### Household socio-demographic factors.

Four household hygiene-related factors showed significant univariable associations with stunting. Having a handwashing place near the toilet and washing with soap in the past 24 hours were associated with lower odds (OR = 0.23; p = 0.002 and OR = 0.51; p = 0.013, respectively). While, handwashing before food preparation only rarely and having a non-improved toilet were associated with higher odds of stunting. Sex of the household head or mother’s relationship with the household head were not statistically significant. Individually, these predictors explained only a small proportion of the variance in stunting (pseudo R^2^ ≤ 0.024).

#### Household economic factors.

Household with access to electricity and household food insecurity (HFIAS score) showed the strongest economic associations with stunting. Children from households with electricity had approximately 49% lower odds of stunting, and electricity access accounted for about 1.7% of stunting variation (pseudo R^2^ = 0.017). Additional moderately significant economic predictors included home garden ownership and no consumption of fresh or fermented milk. Other economic factors such as Ubudehe categories, income levels and house type showed no clear association with stunting (p ≥ 0.1).

#### Child health and nutrition factors.

Child health and nutrition factors associated with higher odds of stunting included older child age, child sex, underweight status and greater distance to the nearest health facility (OR: 1.32–8.70; p < 0.007). Birthweight and current breastfeeding also showed significant associations (OR: 0.55–0.65, p ≤ 0.010). Other predictors, such as having diarrhoea in the last two weeks and consumed types of food demonstrated moderate or marginal significance, respectively.

#### Childcare practices and presence of violence against children.

Childcare-related predictors including receiving deworming tablets (OR = 4.08; p < 0.001), vitamin A supplementation (OR = 3.20; p < 0.001) within the past six months and being fed by someone other than parents in the past two weeks (OR = 1.30; p < 0.007) were associated with higher odds of stunting, while the use of multiple micronutrient powders showed marginal significance. Factors related to violent discipline towards the index child such as shouting, name-calling, hitting with a bare hand or with an object were associated with stunting (OR:1.78 − 2.20; p ≤ 0.012).

#### Maternal health and presence of violence against mothers.

Maternal height and number of antenatal care visits showed only marginal associations (p ≤ 0.086). Prior miscarriages were significantly associated with increased odds of stunting. Lower social support; specifically, absence of a friend to assist when ill and lack of someone to provide guidance in problems, was associated with higher odds of stunting (OR = 1.56; p = 0.058 and OR= 2.03; p = 0.005). Several decision-making autonomy factors such as major purchases, visiting family, and use of earned money also showed higher odds (OR: 3.19 − 4.36; p ≤ 0.008), whereas the ability to say no to sexual intercourse had lower odds (OR = 0.52; p = 0.003). Predictors related to violence against mothers were analysed separately in another paper, part of this broader research initiative [33].

#### Milk production and animal husbandry factors.

Milk production and dairy cow husbandry factors generally showed weak or no statistically significant associations with stunting (S7 Table). Only reproduction technique and the milking place demonstrated marginal significance. Other dairy-related factors, including feeding system, milk production purpose, knowledge about disease transmission, and daily milk yield, showed very limited or no associations (p > 0.2; S7 Table).

#### Environmental physical factors.

The main environmental predictors significantly associated with stunting were distance to the nearest market and higher elevation (OR = 0.27; p = 0.010). The mean annual temperature showed a marginal inverse association (p = 0.058), and other variables were not significant.

### Adjusted associations from a multivariable logistic regression

Statistically significant associations were observed between childhood stunting and multiple predictors across individual, household, and environmental factors. Following feature selection, 24 predictor variables were retained for inclusion in the multivariable logistic regression model. The adjusted model, fitted on 480 observations from the training dataset, accounted for 34% of the variance in stunting outcomes (pseudo R² = 0.34; log-likelihood = −183.86; AIC = 417.71), indicating a considerable improvement in explanatory power compared to univariate models. Adjusted odds ratios and 95% CIs for selected predictors are presented in Table 1.

**Table 1:** Adjusted odds ratios for selected predictors of child stunting from the multivariable logistic regression.

Predictor variables	Adjusted OR (95% CI)	p-value
Child age	2.46 (1.78-3.39)	<0.001
Underweight status	13.40 (4.35-41.26)	<0.001
Child sex (Male)	2.83 (1.65-4.86)	<0.001
Birthweight	0.71 (0.54-0.94)	0.016
Days cared for by another child	0.72 (0.55-0.95)	0.019
Days left alone >1 hour	0.75 (0.56-1.00)	0.053
Reading to the child (yes)	1.86 (1.03-3.35)	0.038
Times child fed by others	1.26 (0.96-1.65)	0.090
Shaking children	0.41 (0.20-0.83)	0.014
Types of food consumed	1.46 (1.06-2.01)	0.022
Milk consumption (none)	1.56 (0.87-2.80)	0.139
Distance to nearest health centre (m)	1.29 (0.98-1.69)	0.072
Elevation (m)	1.53 (1.15-2.03)	0.003
Handwashing facility (yes)	0.21 (0.07-0.67)	0.008
Household electricity access (yes)	0.48 (0.27-0.84)	0.011
Household Food Insecurity (HFIAS)	1.30 (0.98-1.73)	0.065
Education level (secondary, not complete)	1.96 (0.94-4.08)	0.074
Number of prior miscarriages	1.42 (1.09-1.84)	0.009
Number of ANC visits	0.77 (0.59-1.01)	0.059
Number born by caesarean	1.24 (0.96-1.59)	0.097
Ability to refuse sexual intercourse (yes)	0.48 (0.27-0.86)	0.014
Maternal social support (sometimes has help)	2.30 (1.20-4.42)	0.012
Mother’s headache (yes)	2.16 (1.18-3.97)	0.013
Alcohol before pregnancy (seldom/never)	1.80 (1.00-3.22)	0.049

Among child characteristics, increasing age (Adjusted OR = 2.46; 95% CI: 1.78–3.39; p < 0.001), underweight status (OR = 13.40; 95% CI: 4.35–41.26; p < 0.001), and male sex (OR = 2.83; 95% CI: 1.65–4.86; p < 0.001) were positively associated with stunting, while higher birthweight was inversely associated (OR = 0.71; 95% CI: 0.54–0.94; p = 0.016). Within childcare practices, index-child whose mother reported shaking children or children cared for by another child (OR = 0.41 − 0.72; 95% CI: 0.20–0.95; p ≤ 0.019) had lower odds of stunting. Higher elevation and distance to nearest health facility were also positively associated with stunting. Household-level factors such as the presence of a handwashing facility near the toilet and access to electricity were significantly associated with reduced odds, while lack of milk consumption exhibited higher odds. Maternal history of prior miscarriages, maternal autonomy to refuse sexual intercourse, and psychosocial factors such as maternal social support, headache, and alcohol use before pregnancy were also significantly associated with stunting. Several variables, including maternal education, antenatal care visits, and household food insecurity, showed borderline or non-significant associations.

The multivariable logistic regression model demonstrated higher performance on the training dataset, with AUROC = 0.87 and PR-AUC = 0.77, while performance declined (AUROC = 0.70, PR-AUC = 0.58) on the test dataset. Confusion matrix analysis showed that in the training set, 101 stunted and 290 non-stunted children were correctly classified, while 60 non-stunted and 29 stunted cases were misclassified. In the test set, the model correctly identified 18 stunted and 60 non-stunted children, with 28 non-stunted and 15 stunted cases misclassified (sensitivity = 0.78; specificity = 0.83). The village-level mixed-effects sensitivity analysis yielded similar direction and comparable magnitude of the main associations to the global model (S10 Table), indicating that the primary inferences are not driven solely by village-level clustering.

### Fine-scale spatial heterogeneity of stunting risk factors

The GWLR model was calibrated using an adaptive bisquare kernel and a bandwidth of 479 nearest neighbours. It included 22 selected predictor variables and was estimated on 480 observations in the training dataset. Two variables; the number of children born by caesarean and the lack of household milk consumption; were excluded from the spatial analysis after their local coefficients showed near-zero lower bounds. The percent deviance explained was 39.2% (percent deviance = 0.392; log-likelihood =−170.3), an improvement over the global logistic regression model, at the cost of additional model complexity (AIC = 419.03; AICc = 426.18). The adjusted 95% significance threshold for local parameters was α = 0.029 (critical t = 2.185).

Local parameter estimates varied across the study area for multiple factors in both magnitude and direction (Table 2). Child age (mean coefficient = 0.939; SD = 0.139; range: 0.716–1.138) and male sex (mean = 0.991; SD = 0.072; 0.845–1.238) showed consistently positive associations across locations. In contrast, higher birthweight, access to electricity, handwashing facilities, maternal decision autonomy and children being left alone or with minors exhibited negative associations. Elevation, household food insecurity, and distance to health facilities were also positively but moderately associated with stunting. Caregiving and social support indicators (such as child fed by others, reading to the child, and inconsistent maternal support) showed positive associations with moderate spatial variability.

**Table 2:** Summary statistics for the geographically weighted logistic regression parameter estimates.

Predictor variables	Mean	SD	Minimum	Median	Maximum
Intercept	−2.273	0.480	−2.911	−2.325	−1.548
Child age	0.939	0.139	0.716	0.947	1.138
Underweight status	2.734	0.345	2.298	2.644	3.323
Child sex (Male)	0.991	0.072	0.845	0.994	1.238
Birthweight	−0.433	0.078	−0.725	−0.413	−0.34
Days cared for by another child	−0.351	0.013	−0.384	−0.349	−0.317
Days left alone >1 hour	−0.307	0.076	−0.562	−0.286	−0.201
Reading to the child (yes)	0.733	0.208	0.41	0.751	0.995
Times child fed by others	0.251	0.053	0.157	0.247	0.407
Shaking children	−0.947	0.186	−1.325	−0.942	−0.679
Types of food consumed	0.375	0.056	0.269	0.397	0.503
Distance to nearest health centre (m)	0.255	0.048	0.142	0.251	0.366
Elevation (m)	0.421	0.066	0.308	0.421	0.628
Handwashing facility (yes)	−1.785	0.092	−2.112	−1.772	−1.608
Household electricity access (yes)	−0.841	0.166	−1.175	−0.897	−0.54
Household Food Insecurity (HFIAS)	0.301	0.089	0.188	0.266	0.452
Education level (secondary, not complete)	0.756	0.155	0.448	0.814	0.999
Number of prior miscarriages	0.369	0.067	0.248	0.379	0.534
Number of ANC visits	−0.267	0.073	−0.442	−0.259	−0.15
Ability to refuse sexual intercourse (yes)	−0.766	0.201	−1.029	−0.824	−0.409
Maternal social support (sometimes has help)	0.954	0.299	0.456	1.046	1.299
Mother’s headache (yes)	0.874	0.06	0.787	0.868	0.982
Alcohol before pregnancy (seldom/never)	0.758	0.334	0.191	0.883	1.148

**Fig 4:**
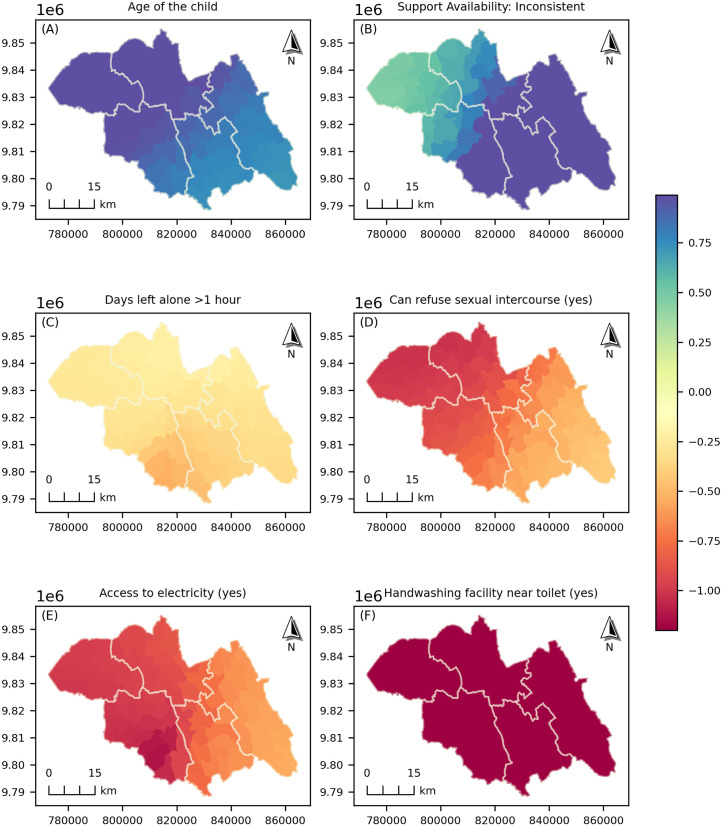
The spatial variation of the local GWLR coefficient estimates for selected predictors across the Northern Province. Maps were generated in Python (GeoPandas/Matplotlib) using model outputs and administrative boundary polygons (Rwanda MININFRA GeoPortal).

**Fig 5:**
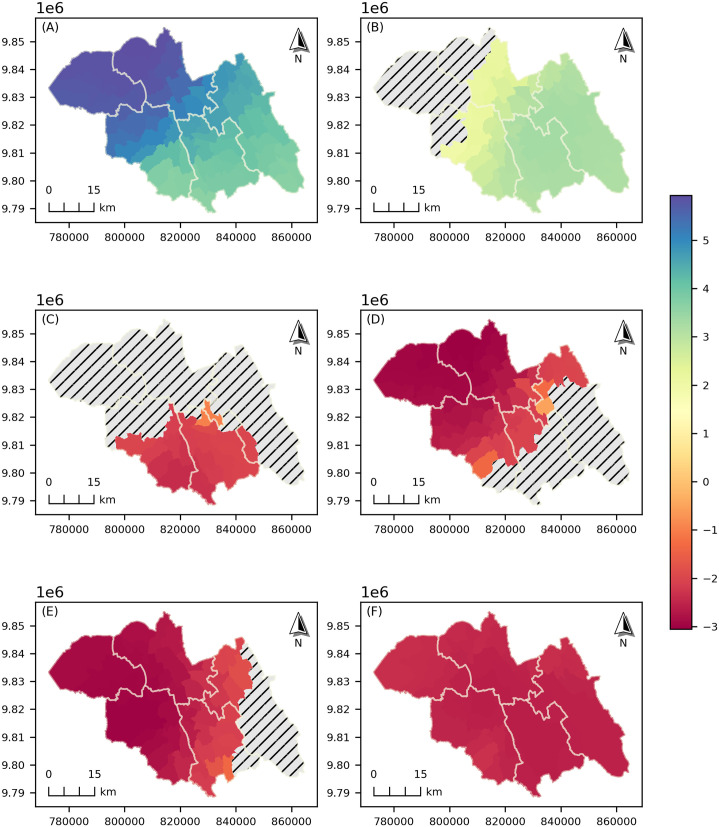
Spatial distribution of local t-values of GWLR coefficients (for selected predictors). Statistically non-significant areas (α = 0.05; |*t*| < 1.96) are indicated with hatched patterns. Maps were generated in Python (GeoPandas/Matplotlib) using GWLR outputs and administrative boundary polygons (Rwanda MININFRA GeoPortal).

Fig 4 presents the spatial distribution of local GWLR parameter estimates for six selected variables, showing spatial heterogeneity in the predictors of childhood stunting across the study area. These mapped variables were not only chosen based on statistical influence but rather for their relevance to policy formulation and public health interventions. Specifically, they represent domains such as household living conditions, maternal empowerment, and hygiene practices; factors directly linked to actionable programs. Access to electricity and maternal autonomy (measured as the ability to refuse sexual intercourse) show negative associations with varied magnitude, while the presence of handwashing facilities near toilets exhibits a consistently strong-negative spatial association across the study area. Comprehensive maps of all local coefficients are provided in the Supplementary Material (S1 Fig). Fig 5 shows the spatial distribution of local t-values for the six mapped variables, identifying areas where predictors of childhood stunting have statistically significant effects (α = 0.05; |*t*| > 1.96). The corresponding t-value maps for all model variables are provided in the Supplementary Material (S2 Fig).

**Fig 6:**
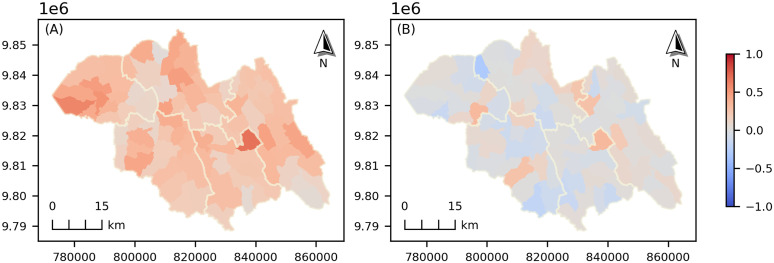
Spatial distribution of (A) predicted probability of child stunting and (B) GWLR residuals. Maps were generated in Python (GeoPandas/Matplotlib) using GWLR outputs and administrative boundary polygons (Rwanda MININFRA GeoPortal).

Fig 6(A) shows the spatial distribution of stunting rates predicted by the GWLR model, highlighting notable spatial heterogeneity across the study area. Fig 6(B) presents the GWLR residuals, which exhibit a random spatial pattern with no discernible clustering. This suggests that the model has effectively accounted for spatial dependence, leaving minimal spatial autocorrelation in the residuals. Moreover, the Moran’s I test for spatial autocorrelation returned a Moran’s Index of 0.018 and a *p*-value of 0.242, indicating no statistically significant spatial autocorrelation in the residuals. Confirming that GWLR model adequately accounted for spatial patterns in the data.

Model diagnostics (S3 Fig) indicated higher performance on the training dataset (AUROC = 0.89; PR AUC = 0.80) but lower out-of-sample performance in the test dataset (AUROC = 0.68; PR-AUC = 0.53). Calibration also declined in the test set, as indicated by greater deviation from the 45° line and a higher Brier score (0.209 vs 0.108 in training).

## Discussion

This cross-sectional study examined fine-scale spatial heterogeneity and interdisciplinary determinants of stunting among children aged 1 − 36 months in the Northern Province of Rwanda. By integrating geocoded household survey data with spatial modelling, we identified both global and localised determinants of stunting and observed improved model fit when allowing coefficients to vary spatially (global logistic regression pseudo R^2^ = 0.34; GWLR deviance explained = 0.392). These findings underscore the limitations of assuming uniform relationships across geographic areas and support geographically targeted interventions.

Traditional reliance on national-level datasets such as the DHS, while valuable for monitoring, constrains fine-scale spatial analysis due to data aggregation and spatial displacement [11,12,52]. By using household-level geographic data, our study enables the identification of spatially varying relationships between stunting and factors spanning child health, caregiving practices, maternal autonomy, household infrastructure and hygiene conditions. These spatial variations indicate that broad, one-size-fits-all interventions may overlook important local drivers of risk.

At the child level, increasing age, male sex, and concurrent underweight status were strongly associated with stunting, while higher birthweight was protective. These patterns align with evidence from Rwanda and other low-resource settings that linear growth faltering accelerates after infancy and is compounded by early-life growth constraints and morbidity [11–13,17]. The strong association between underweight and stunting likely reflects shared underlying drivers rather than a direct causal pathway, underscoring the importance of integrated programming that addresses both chronic and acute forms of undernutrition.

At the household and community level, the presence of a handwashing facility near the toilet and household electricity access were consistently associated with lower odds of stunting, although the magnitude of these associations varied geographically in the GWLR models. These indicators plausibly operate as both direct determinants (through reduced pathogen exposure and improved hygiene behaviours) and proxies for broader socioeconomic advantage that influences diet quality, caregiving, and health service utilisation [13,14,53,54]. The spatially varying coefficients suggest that WASH and infrastructure investments may yield disproportionate nutritional benefits in specific local contexts, supporting the use of granular maps for prioritisation.

Maternal autonomy (ability to refuse sexual intercourse) was inversely associated with child stunting, consistent with literature linking women’s empowerment to improved child feeding, healthcare seeking, and household resource allocation [55]. In contrast, inconsistent maternal social support was associated with higher odds of stunting, highlighting the potential role of psychosocial stress and reduced caregiving capacity in pathways leading to growth faltering. The observed spatial heterogeneity indicates that interventions strengthening women’s agency and community-based support structures may be particularly impactful in areas where these associations are strongest [56,57].

Several caregiving and service-related indicators showed complex or counterintuitive associations in bivariate analyses (such as deworming tablets or vitamin A supplementation correlating with higher odds of stunting), which likely reflect programme targeting or reverse causality; children in poorer health may be more likely to receive services. Similarly, inverse associations observed for some self-reported caregiving practices should be interpreted cautiously due to potential reporting bias and residual confounding. These findings reinforce the need to interpret cross-sectional associations in light of programme context and to prioritise longitudinal designs for causal inference [58].

Although dairy cow husbandry variables were not strongly associated with stunting in the adjusted models, these findings do not negate the broader “One Health” relevance of livestock systems for child nutrition. Animal-source foods can improve dietary quality, while zoonotic exposures and food safety practices may indirectly influence child growth through infection and inflammation pathways [59–61]. Future analyses combining nutritional intake, microbiological outcomes, and longitudinal follow-up could better characterise these indirect mechanisms.

A key methodological contribution of this study is the use of GWLR to relax the assumption that risk-factor relationships are spatially stationary. Importantly, the inclusion of predictors from different conceptual domains (child, household, environment) reflects the multifactorial pathways to stunting. A village-level mixed-effects logistic regression sensitivity analysis showed broadly consistent fixed-effect associations (S10 Table), indicating that the main inferences are not driven only by intra-village correlation. Our primary objective was to characterise spatially continuous heterogeneity across the landscape; mixed-effects models address hierarchical clustering but do not provide spatially varying coefficients. Caution is also warranted when interpreting predictive performance: although model fit was strong in the training data, discrimination declined in the held-out dataset, so results should be interpreted primarily for spatial inference rather than individual-level prediction. Finally, the cross-sectional design limits causal inference [58], and residual confounding may persist due to unmeasured local factors such as market access [62], healthcare quality, seasonal food availability, economic opportunities, or environmental exposures (such as soil-transmitted helminths) [63,64]. Longitudinal and intervention-based studies are needed to validate and extend these findings.

## Conclusion

This study provides strong evidence that childhood stunting in Northern Rwanda results from a complex interplay of socio-demographic, economic, environmental, and health-related factors exhibiting significant spatial variability. Through interdisciplinary collaboration and advanced spatial modelling, we identified geographically distinct risk patterns that could not have been captured by conventional analytical methods alone. The use of GWLR was particularly valuable in identifying location-specific factors, offering a powerful methodological approach for other studies in global public health.

Our findings suggest critical policy implications, emphasizing the importance of context-specific, targeted interventions. Strategies such as enhancing maternal empowerment and support systems, and improving household infrastructure (particularly sanitation facilities) can substantially address childhood stunting. Localised interventions should be prioritized to address specific needs identified in geographically distinct clusters, optimizing resource allocation and intervention effectiveness.

This spatially explicit approach supports the strategic allocation of limited resources, particularly critical in resource-constrained settings with persistent stunting prevalence. Future studies should build upon such methodological advances, exploring longitudinal data to confirm causal relationships and validate spatially targeted interventions to enhance child nutritional outcomes sustainably.

## Supporting information

S1 Checklist STROBE statement. (DOCX)

S1 Data The minimal anonymised dataset. (CSV)

S2 Data The variable codebook (variable description). (CSV)

S1 Fig Spatial distribution of local coefficient estimates from GWLR model. Each map illustrates the spatial variation in the estimated effect of a predictor on childhood stunting across the study area.
(TIF)

S2 Fig Spatial distribution of local *t*-values of GWLR coefficients. Areas where coefficients are not statistically significant (α = 0.05; |*t*| < 1.96) are indicated with hatched patterns.
(TIF)

S3 Fig Model performance diagnostics for GWLR. (A) Receiver Operating Characteristic (ROC) curves for the GWLR model. (B) Calibration plots for predicted stunting probabilities, with the dashed green line represents perfect calibration.
(TIF)

S1 Table Summary statistics of household socio-demographic factors. (DOCX)

S2 Table Summary statistics of household economic factors. (DOCX)

S3 Table Summary statistics of child health and nutrition factors. (DOCX)

S4 Table Summary statistics of the childcare practice factors. (DOCX)

S5 Table Summary statistics of violence against children factors. (DOCX)

S6 Table Summary statistics of maternal characteristics. (DOCX)

S7 Table Summary statistics of dairy production and animal health factors. (DOCX)

S8 Table Summary statistics of environmental physical factors. (DOCX)

S9 Table Summary statistics for selected predictor-variables from univariable logistic regression model. (DOCX)

S10 Table Sensitivity analysis: mixed-effects logistic regression with village random intercept for predictors of childhood stunting. (DOCX)
