# Supplementary material for: Spatial heterogeneity and spatially varying determinants of childhood stunting in Northern Rwanda: A cross-sectional study to inform targeted interventions
Source: PLoS One. 2026 Feb 26;21(2):e0343772. doi: 10.1371/journal.pone.0343772 (PMC12944770; doi:10.1371/journal.pone.0343772)
Supplement: S1 Checklist — (DOCX) [file pone.0343772.s001.docx]

**S1 Checklist: STROBE Statement**

Checklist of items that should be included in reports of cross-sectional studies

|  | Item No | Recommendation | Page No |
| --- | --- | --- | --- |
| **Title and abstract** | 1 | (*a*) Indicate the study’s design with a commonly used term in the title or the abstract | Page 1-2: *Title and Abstract* |
|  |  | (*b*) Provide in the abstract an informative and balanced summary of what was done and what was found | Pages 2-3: *Abstract* |
| Introduction | | | |
| Background/rationale | 2 | Explain the scientific background and rationale for the investigation being reported | Pages 3-6: *Background* |
| Objectives | 3 | State specific objectives, including any prespecified hypotheses | Page 6 (From Line 110 - 122):  *Background (last paragraphs)* |
| Methods | | | |
| Study design | 4 | Present key elements of study design early in the paper | Page 6-7: *Study design and study area* |
| Setting | 5 | Describe the setting, locations, and relevant dates, including periods of recruitment, exposure, follow-up, and data collection | Pages 6-7: *Study design and study area* |
| Participants | 6 | (*a*) Give the eligibility criteria, and the sources and methods of selection of participants | Page 10 (Line 193 - 200)*: Study participants’ enrolment* |
| Variables | 7 | Clearly define all outcomes, exposures, predictors, potential confounders, and effect modifiers. Give diagnostic criteria, if applicable | Page 12 (Lin 226 - 239): *Outcome variables*  Page 12-15: *Potential predictors of childhood stunting* |
| Data sources/ measurement | 8* | For each variable of interest, give sources of data and details of methods of assessment (measurement). Describe comparability of assessment methods if there is more than one group | - Page 7 -8 (Line 139 - 152):   *Questionnaire data and geographic coordinates*   - Page 10 (Line 201 - 207):   *Anthropometric measurements*   - Page 11 (Line 208-225):   *Blood and stool sample collection*   - Page 15 (line 287-296)*:*   *Environmental data (existing data)* |
| Bias | 9 | Describe any efforts to address potential sources of bias | Efforts to address potential sources of bias were described in the *Methods section*:   - *Systematic random sampling*   *Page 8-9 ( Line 155 - 178)*:  Use of a two-stage cluster random sampling design ensuring geographic representativeness and systematic random selection of households within clusters.   - *Measurements bias (Page 9-11, Line 179 - 225)*: - Training of enumerators using standardised protocols to ensure consistent data collection (anthropometric measures, questionnaires, laboratory samples). - The use of validated questionnaires and standardised measurement tools (UNICEF height boards, digital scales, standardised laboratory methods). - *Data collection bias (Page 7-9)*:   *Lines: 139-144;149 – 151; 189-192*  Pilot study and real-time data collection using a GPS-enabled mobile GIS platform allowed to minimise errors in data recording and facilitate immediate quality control.   - *Missing data bias (Page 15-16, Lines: 306-310)*:   Clear handling of missing data. |
| Study size | 10 | Explain how the study size was arrived at | Page 8-9 (Line 155-178)  *Methods (Community and demographic based sampling)* |
| Quantitative variables | 11 | Explain how quantitative variables were handled in the analyses. If applicable, describe which groupings were chosen and why | Page 16- 20:  *Methods:*  *Data analysis:*  *Exploratory data analysis*  *Spatial statistical analysis* |
| Statistical methods | 12 | (*a*) Describe all statistical methods, including those used to control for confounding | Page 16-19: *Methods*  Both multivariable logistic regression and geographically weighted logistic regression (GWLR) were used and described in the methods section. |
|  |  | (*b*) Describe any methods used to examine subgroups and interactions | Page 17-19: *Methods*  GWLR was used to explore spatially varying relationships (local spatial interactions). However, in the multivariable logistic regression, interaction effects between variables were not explicitly assessed. |
|  |  | (*c*) Explain how missing data were addressed | Page 15-16 (Lines: 304-310): *Methods (Data pre-processing)* |
|  |  | (*d*) If applicable, describe analytical methods taking account of sampling strategy | Page 8-9 (Lines: 155-178) |
|  |  | (*e*) Describe any sensitivity analyses | Sensitivity analyses were not conducted in this study |
| Results | | | |
| Participants | 13* | (a) Report numbers of individuals at each stage of study—eg numbers potentially eligible, examined for eligibility, confirmed eligible, included in the study, completing follow-up, and analysed | Page 16(Line 306-307)  The final analysis included 601 households, selected from an initial sample size of 615 households. Fourteen records (2%) were excluded due to extensive missing data. |
|  |  | (b) Give reasons for non-participation at each stage | Page 10:   - Households with mothers younger than 18 years, unavailable mothers, or mothers too ill to participate were excluded according to predefined inclusion/exclusion criteria. |
|  |  | (c) Consider use of a flow diagram | A participant flow diagram was not included in the manuscript. |
| Descriptive data | 14* | (a) Give characteristics of study participants (eg demographic, clinical, social) and information on exposures and potential confounders | Page 10 (Line 193-200) & Page 13-14: |
|  |  | (b) Indicate number of participants with missing data for each variable of interest | Page 15 (line 306-307): *Methods (Data pre-processing);*  Page 21 - 24: *Results (More details on descriptive statistics, including missing data, are in Supplementary Material: Tables S1-S8 in S1 File)* |
| Outcome data | 15* | Report numbers of outcome events or summary measures | Page 21: *Results (Stunting, wasting, and underweight: sex- and district-specific patterns)*   - The main outcome was stunting (height-for-age z-score < -2 SD). The study reported that out of the 601 children included in the final analysis, 27% were stunted. - Other nutritional outcomes reported were wasting (weight-for-height z-score < -2 SD) at 3% and underweight (weight-for-age z-score < -2 SD) at 7%. |
| Main results | 16 | (*a*) Give unadjusted estimates and, if applicable, confounder-adjusted estimates and their precision (eg, 95% confidence interval). Make clear which confounders were adjusted for and why they were included | Page 22-25: *Results*  More details clearly reported in Supplementary Materials:   - Unadjusted (crude) associations between stunting and each individual predictor *(Supplementary Material: Tables S1- S9 in S1 File)*   Page 25-30   - Adjusted estimates (multivariable logistic regression and GWLR) |
|  |  | (*b*) Report category boundaries when continuous variables were categorized | Page 12 (lines: 226-239): *Outcome variables*  Categorization boundaries reported for nutritional outcomes (stunting defined as height-for-age z-score < -2 SD, wasting as weight-for-height z-score < -2 SD, and underweight as weight-for-age z-score < -2 SD. |
|  |  | (*c*) If relevant, consider translating estimates of relative risk into absolute risk for a meaningful time period | N/A |
| Other analyses | 17 | Report other analyses done—eg analyses of subgroups and interactions, and sensitivity analyses | Page 18-19: *Methods (Spatial statistical analysis)*  Page 27-30: *Results (from GWLR model)*  GWLR analysis provided geographically specific coefficient estimates. |
| Discussion | | | |
| Key results | 18 | Summarise key results with reference to study objectives | *Results*  - Page 21: Lines 412, 417 - 419  - Page 28: Lines: 526 – 528; 531-532 |
| Limitations | 19 | Discuss limitations of the study, taking into account sources of potential bias or imprecision. Discuss both direction and magnitude of any potential bias | *Discussion*  Page 32 (Lines: 609-611; 615-619) |
| Interpretation | 20 | Give a cautious overall interpretation of results considering objectives, limitations, multiplicity of analyses, results from similar studies, and other relevant evidence | *Discussion*  Page 33 (line 615-619): |
| Generalisability | 21 | Discuss the generalisability (external validity) of the study results | Page 33-34: *Discussion*  Lines: 579-603 |
| Other information | | | |
| Funding | 22 | Give the source of funding and the role of the funders for the present study and, if applicable, for the original study on which the present article is based | *Funding information*   - The study was funded by the Swedish International Development Cooperation Agency (SIDA), under SIDA contribution No. 11277. - The funders had no role in the design, data collection, analysis, interpretation of the results, or manuscript writing. |

*Give information separately for exposed and unexposed groups: *Not applicable*.
