# Supplementary figures and images for: Spatial heterogeneity and spatially varying determinants of childhood stunting in Northern Rwanda: A cross-sectional study to inform targeted interventions
Source: PLoS One. 2026 Feb 26;21(2):e0343772. doi: 10.1371/journal.pone.0343772 (PMC12944770; doi:10.1371/journal.pone.0343772)

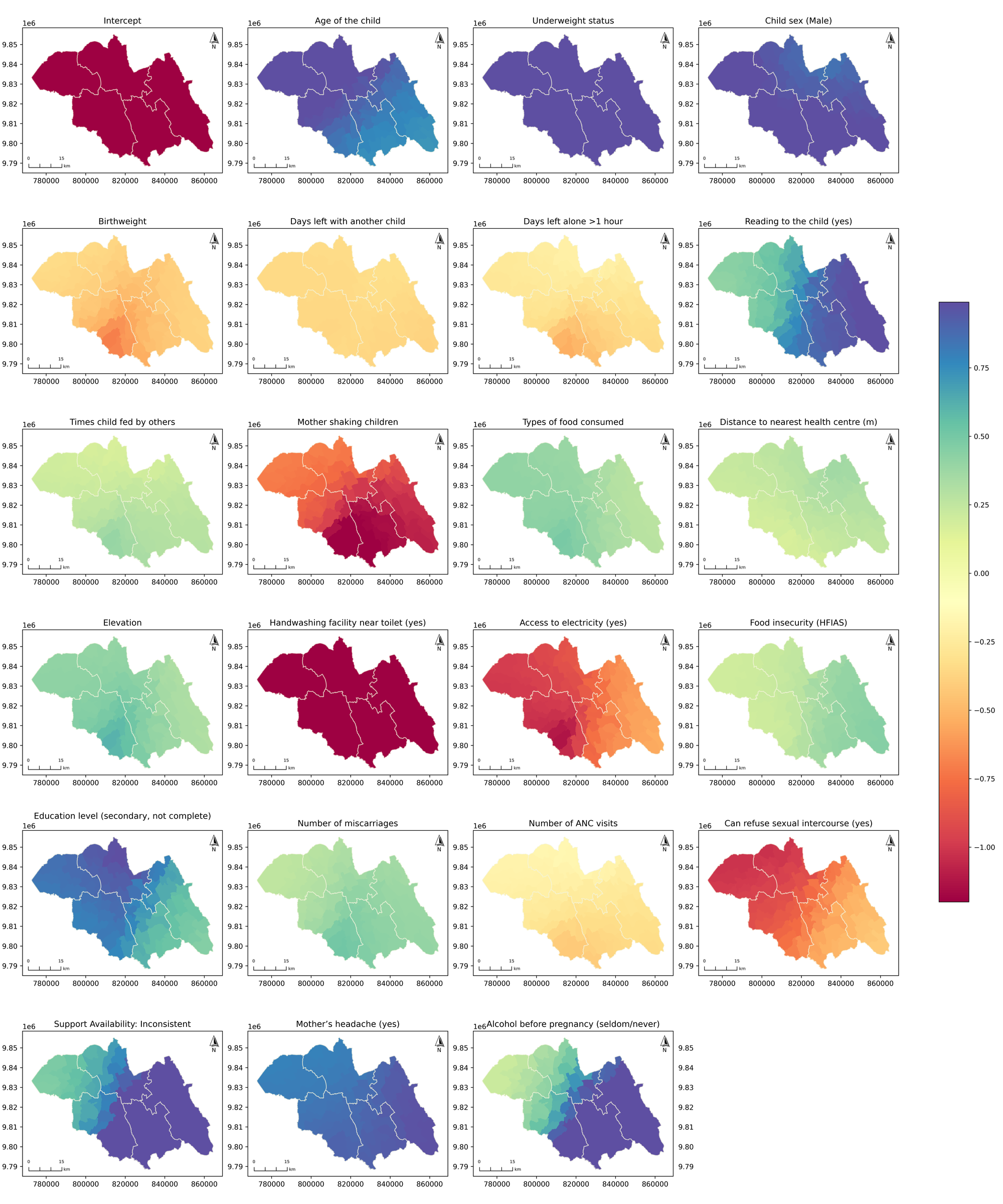

Supplement: S1 Fig — Each map illustrates the spatial variation in the estimated effect of a predictor on childhood stunting across the study area. (TIF) [file pone.0343772.s004.tif]

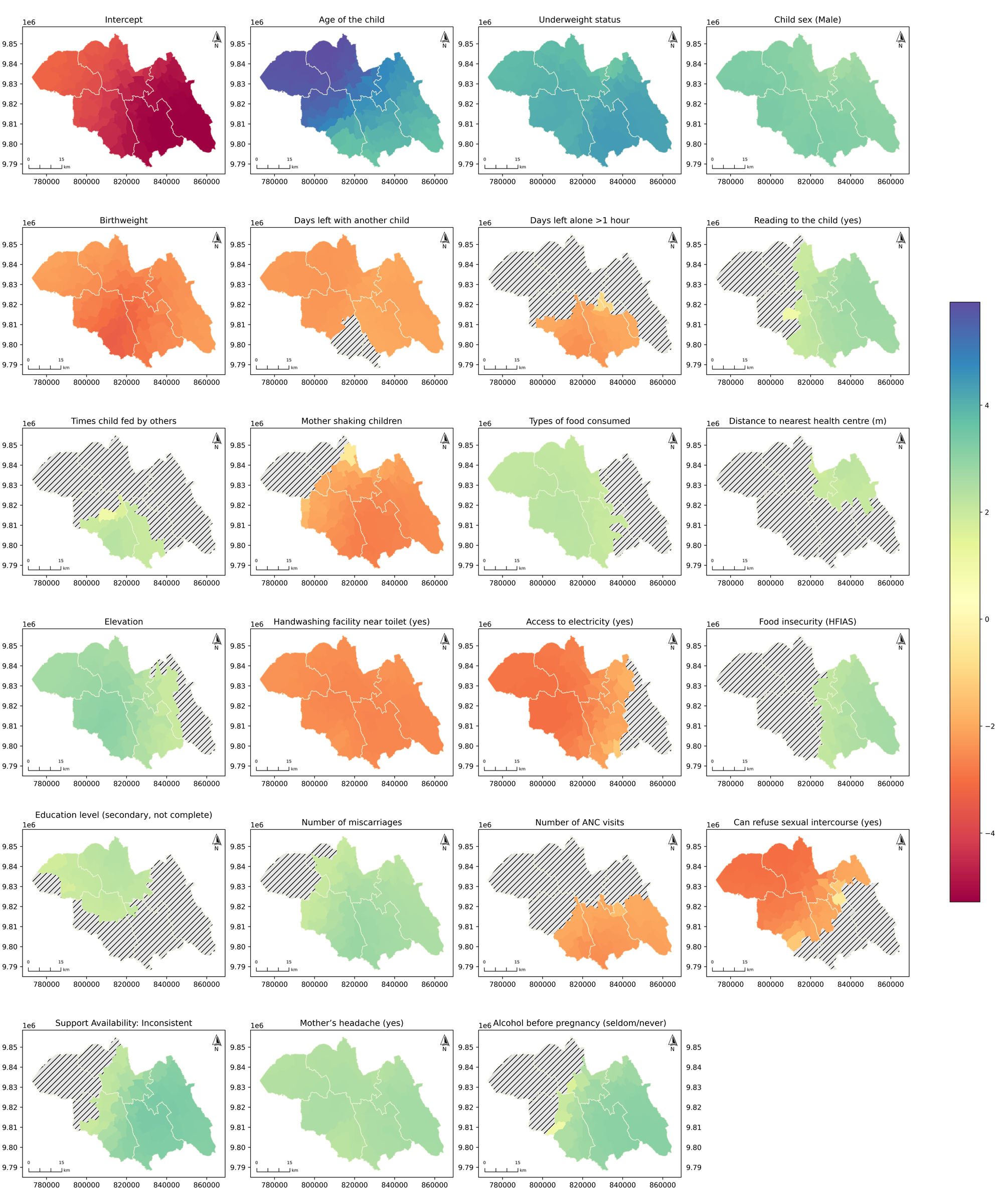

Supplement: S2 Fig — Areas where coefficients are not statistically significant (α = 0.05; |t| < 1.96) are indicated with hatched patterns. (TIF) [file pone.0343772.s005.tif]

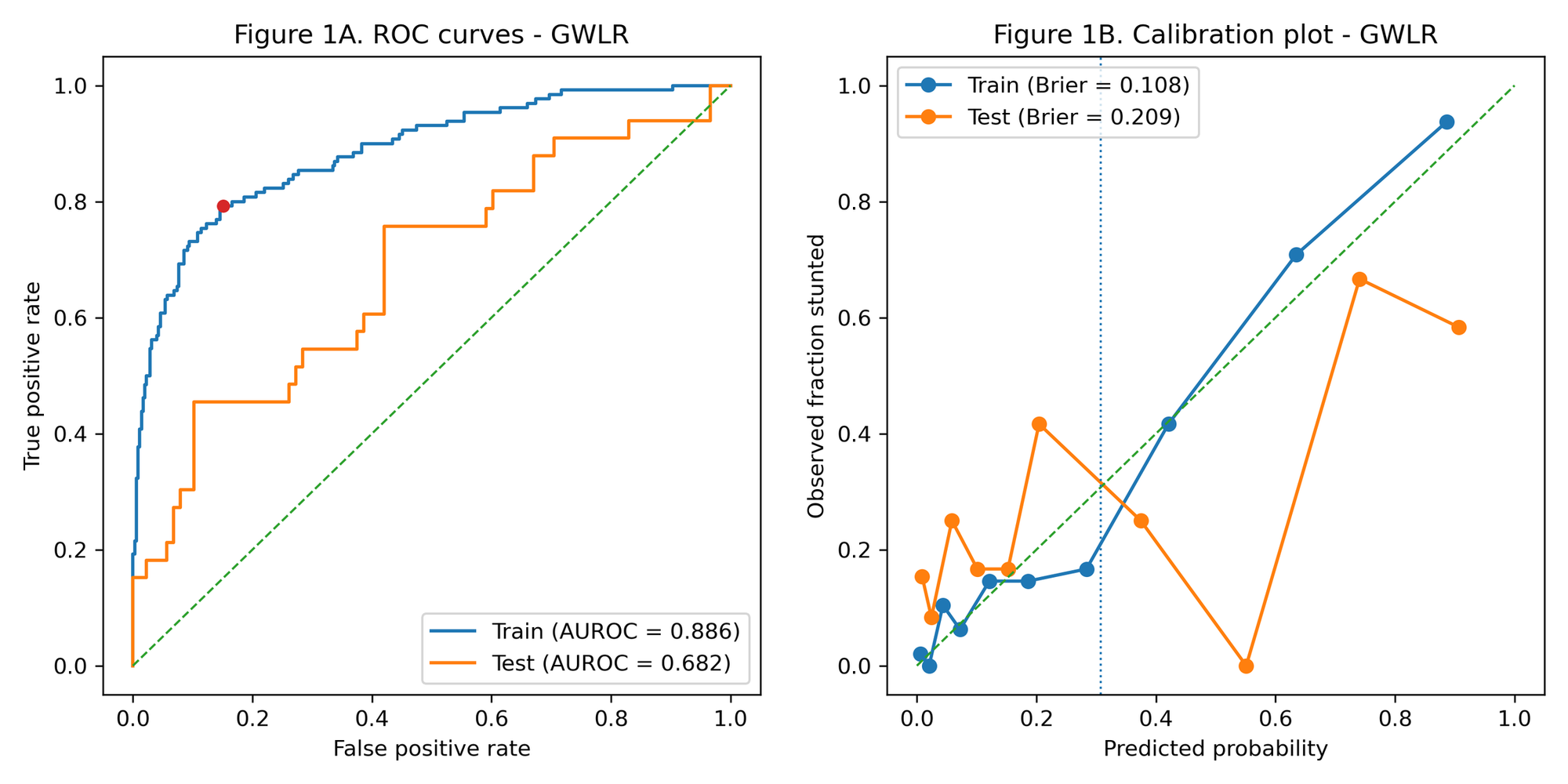

Supplement: S3 Fig — (A) Receiver Operating Characteristic (ROC) curves for the GWLR model. (B) Calibration plots for predicted stunting probabilities, with the dashed green line represents perfect calibration. (TIF) [file pone.0343772.s006.tif]
