# Supplementary material for: Spatial heterogeneity and spatially varying determinants of childhood stunting in Northern Rwanda: A cross-sectional study to inform targeted interventions
Source: PLoS One. 2026 Feb 26;21(2):e0343772. doi: 10.1371/journal.pone.0343772 (PMC12944770; doi:10.1371/journal.pone.0343772)
Supplement: S1 Table — (DOCX) [file pone.0343772.s007.docx]

S1 Table. Summary statistics of household socio-demographic factors

| - Descriptive statistics are stratified by child stunting status (not-stunted N=438; stunted N=163) - N: total number of non‑missing observations. Values are n (%) for categorical variables (percent of non-missing observations, across both strata). - ^1^Pearson’s Chi-squared tests or Fisher’s exact test; Statistical significance was evaluated at α = 0.05. | | | | |
| --- | --- | --- | --- | --- |
| **SOCIO-DEMOGRAPHIC FACTORS** | **N** | **Stunting status** | | **p-value**^1^ |
|  |  | **Not-stunted**, *n (%)* | **Stunted**, *n / N (%)* |  |
| District | 601 |  |  | 0.5 |
| Burera |  | 90 (15%) | 39 (6.5%) |  |
| Gakenke |  | 96 (16%) | 34 (5.7%) |  |
| Gicumbi |  | 108 (18%) | 36 (6%) |  |
| Musanze |  | 65 (10.8%) | 31 (5.2%) |  |
| Rulindo |  | 79 (13.1%) | 23 (3.8%) |  |
| Sex of household head | 600 |  |  | 0.014 |
| Female |  | 27 (4.500%) | 20 (3.333%) |  |
| Male |  | 410 (68.33%) | 143 (23.83%) |  |
| Missing |  | 1 | 0 |  |
| Mother's relationship with household head | 601 |  |  | 0.3 |
| Daughter |  | 32 (5.324%) | 14 (2.329%) |  |
| I am head of household |  | 11 (1.830%) | 8 (1.331%) |  |
| Other family relation |  | 5 (0.832%) | 3 (0.499%) |  |
| Wife |  | 390 (64.89%) | 138 (22.96%) |  |
| Religion | 601 |  |  | 0.11 |
| Adventist |  | 49 (8.153%) | 25 (4.160%) |  |
| Catholicism |  | 227 (37.77%) | 79 (13.14%) |  |
| Islam |  | 1 (0.166%) | 0 (0%) |  |
| No religion |  | 4 (0.666%) | 5 (0.832%) |  |
| Other religion |  | 17 (2.829%) | 2 (0.333%) |  |
| Protestantism |  | 140 (23.29%) | 52 (8.652%) |  |
| Health insurance | 595 |  |  | 0.7 |
| No |  | 11 (1.849%) | 6 (1.008%) |  |
| Yes, community based |  | 410 (68.91%) | 151 (25.38%) |  |
| Yes, Public |  | 13 (2.185%) | 4 (0.672%) |  |
| Missing |  | 4 | 2 |  |
| Partner attended school | 562 |  |  | 0.3 |
| I don't know |  | 9 (1.601%) | 1 (0.178%) |  |
| No |  | 42 (7.473%) | 20 (3.559%) |  |
| Yes |  | 366 (65.12%) | 124 (22.06%) |  |
| Missing |  | 21 | 18 |  |
| Partner has a job | 560 |  |  | 0.6 |
| No |  | 292 (52.14%) | 99 (17.68%) |  |
| Yes |  | 123 (21.96%) | 46 (8.214%) |  |
| Missing |  | 23 | 18 |  |
| Belongs to an association | 598 |  |  | 0.3 |
| No |  | 153 (25.59%) | 65 (10.87%) |  |
| Yes |  | 283 (47.32%) | 97 (16.22%) |  |
| Missing |  | 2 | 1 |  |
| Drinking water sources | 599 |  |  | 0.6 |
| Improved source |  | 279 (46.58%) | 97 (16.19%) |  |
| Improved source; Unimproved source |  | 6 (1.002%) | 2 (0.334%) |  |
| Other |  | 1 (0.167%) | 0 (0%) |  |
| Unimproved source |  | 150 (25.04%) | 64 (10.68%) |  |
| Missing |  | 2 | 0 |  |
| Treat drinking water | 601 |  |  | 0.6 |
| Don't remember |  | 1 (0.166%) | 0 (0%) |  |
| No |  | 233 (38.77%) | 93 (15.47%) |  |
| Yes |  | 204 (33.94%) | 70 (11.65%) |  |
| Kind of toilet | 601 |  |  | 0.047 |
| Improved |  | 209 (34.78%) | 63 (10.48%) |  |
| Non-improved |  | 229 (38.10%) | 100 (16.64%) |  |
| Washed with soap the last 24h | 599 |  |  | 0.014 |
| No |  | 52 (8.681%) | 32 (5.342%) |  |
| Yes |  | 385 (64.27%) | 130 (21.70%) |  |
| Missing |  | 1 | 1 |  |
| Have a handwashing place near the toilet | 601 |  |  | <0.001 |
| No |  | 376 (62.56%) | 156 (25.96%) |  |
| Yes |  | 62 (10.32%) | 7 (1.165%) |  |
| Washes hand before preparing food | 598 |  |  | 0.050 |
| Always |  | 274 (45.82%) | 89 (14.88%) |  |
| Never |  | 5 (0.836%) | 5 (0.836%) |  |
| Rarely |  | 27 (4.515%) | 18 (3.010%) |  |
| Sometimes |  | 130 (21.74%) | 50 (8.361%) |  |
| Missing |  | 2 | 1 |  |
