# Supplementary material for: Spatial heterogeneity and spatially varying determinants of childhood stunting in Northern Rwanda: A cross-sectional study to inform targeted interventions
Source: PLoS One. 2026 Feb 26;21(2):e0343772. doi: 10.1371/journal.pone.0343772 (PMC12944770; doi:10.1371/journal.pone.0343772)
Supplement: S2 Table — (DOCX) [file pone.0343772.s008.docx]

S2 Table. Summary statistics of household economic factors

| - Descriptive statistics are stratified by child stunting status (not-stunted N=438; stunted N=163) - N: total number of non‑missing observations; Values are n (%) for categorical variables (percent of non-missing observations, across both strata); Continuous variables are summarised as Median (IQR) and Mean (SD) with observed range.   - IQR: Interquartile range, SD: standard deviation - ^1^Pearson’s Chi-squared tests or Fisher’s exact test; Wilcoxon rank‑sum (continuous). Statistical significance was evaluated at α = 0.05. | | | | |
| --- | --- | --- | --- | --- |
| **ECONIMIC FACTORS** | ***N*** | ***Stunting status*** | | ***p-value****^1^* |
|  |  | **Not-stunted**, *n (%)* | **Stunted**, *n (%)* |  |
| Ubudehe categories | 601 |  |  | 0.7 |
| Ubudehe 2 |  | 208 (47.49%) | 84 (51.53%) |  |
| Ubudehe 3 |  | 180 (41.10%) | 61 (37.42%) |  |
| Ubudehe 1 |  | 47 (10.73%) | 16 (9.816%) |  |
| No category given |  | 3 (0.685%) | 2 (1.227%) |  |
| Total household income | 601 |  |  | >0.9 |
| Less than 17,500 RWF |  | 247 (56.39%) | 93 (57.06%) |  |
| 17,500-35,000 RWF |  | 119 (27.17%) | 41 (25.15%) |  |
| 36,000-99,000 RWF |  | 49 (11.19%) | 22 (13.50%) |  |
| 100,000-499,000 RWF |  | 17 (3.881%) | 6 (3.681%) |  |
| I don't know |  | 5 (1.142%) | 1 (0.613%) |  |
| More than 499,000 RWF |  | 1 (0.228%) | 0 (0%) |  |
| Sold household assets (last month) | 599 |  |  | 0.6 |
| No did not sell |  | 382 (87.61%) | 148 (90.80%) |  |
| Yes, for food and others |  | 21 (4.817%) | 6 (3.681%) |  |
| Yes, other |  | 21 (4.817%) | 4 (2.454%) |  |
| Yes, on health care expenditure |  | 12 (2.752%) | 5 (3.067%) |  |
| Missing |  | 2 | 0 |  |
| Access to electricity | 601 |  |  | <0.001 |
| No |  | 253 (57.76%) | 122 (74.85%) |  |
| Yes |  | 185 (42.24%) | 41 (25.15%) |  |
| Owned transportation means | 593 |  |  | 0.089 |
| None of these |  | 372 (85.91%) | 143 (89.38%) |  |
| Bicycle |  | 53 (12.24%) | 16 (10.00%) |  |
| Motorcycle |  | 8 (1.848%) | 0 (0%) |  |
| Car |  | 0 (0%) | 1 (0.625%) |  |
| Missing |  | 5 | 3 |  |
| Owned assets | 598 |  |  | 0.2 |
| Mattress, Mobile phone |  | 273 (62.61%) | 90 (55.56%) |  |
| Mobile phone |  | 86 (19.72%) | 41 (25.31%) |  |
| None of these |  | 31 (7.110%) | 15 (9.259%) |  |
| Mattress |  | 25 (5.734%) | 13 (8.025%) |  |
| Mattress, Mobile phone, TV |  | 20 (4.587%) | 3 (1.852%) |  |
| Mattress, Mobile phone, TV, Refrigerator |  | 1 (0.229%) | 0 (0%) |  |
| Missing |  | 2 | 1 |  |
| Roof type | 573 |  |  | 0.056 |
| Iron sheet |  | 329 (78.71%) | 133 (85.81%) |  |
| Tiles |  | 89 (21.29%) | 22 (14.19%) |  |
| Missing |  | 20 | 8 |  |
| Wall type | 573 |  |  | 0.046 |
| Cemented wall |  | 221 (52.87%) | 66 (42.58%) |  |
| Wood and mud/shrub |  | 111 (26.56%) | 45 (29.03%) |  |
| Adobe bricks or concrete |  | 75 (17.94%) | 42 (27.10%) |  |
| Burnt bricks |  | 11 (2.632%) | 2 (1.290%) |  |
| Missing |  | 20 | 8 |  |
| Floor type | 573 |  |  | 0.4 |
| Mud |  | 322 (77.03%) | 124 (80.00%) |  |
| Cement |  | 96 (22.97%) | 31 (20.00%) |  |
| Missing |  | 20 | 8 |  |
| Household Food Insecurity Access Scale | 601 |  |  | 0.001 |
| Median (IQR) |  | 9 (3 - 14) | 11 (6 - 17) |  |
| Mean (SD) |  | 10 (7) | 12 (7) |  |
| Range |  | 0 - 27 | 0 - 27 |  |
| Grown nutrients-dense crops | 538 |  |  | 0.6 |
| None |  | 215 (54.16%) | 82 (58.16%) |  |
| Orange-fleshed sweet potatoes |  | 110 (27.71%) | 33 (23.40%) |  |
| Iron-rich beans |  | 72 (18.14%) | 26 (18.44%) |  |
| Missing |  | 41 | 22 |  |
| Own a home garden | 601 |  |  | 0.015 |
| Yes |  | 338 (77.17%) | 110 (67.48%) |  |
| No |  | 100 (22.83%) | 53 (32.52%) |  |
| Type of livestock | 601 |  |  | 0.2 |
| Cattle and other livestock |  | 167 (38.13%) | 48 (29.45%) |  |
| Cattle only |  | 118 (26.94%) | 56 (34.36%) |  |
| Other livestock |  | 86 (19.63%) | 31 (19.02%) |  |
| No livestock |  | 67 (15.30%) | 28 (17.18%) |  |
| Livestock | 601 |  |  | 0.6 |
| Yes |  | 371 (84.70%) | 135 (82.82%) |  |
| No |  | 67 (15.30%) | 28 (17.18%) |  |
| Type of milk product | 598 |  |  | 0.066 |
| No milk |  | 236 (54.13%) | 107 (66.05%) |  |
| Fresh milk & Fermented milk (locally called Ikivuguto) |  | 95 (21.79%) | 26 (16.05%) |  |
| Fresh milk |  | 75 (17.20%) | 19 (11.73%) |  |
| Fermented milk (Ikivuguto) |  | 30 (6.881%) | 10 (6.173%) |  |
| Missing |  | 2 | 1 |  |
