# Supplementary material for: Spatial heterogeneity and spatially varying determinants of childhood stunting in Northern Rwanda: A cross-sectional study to inform targeted interventions
Source: PLoS One. 2026 Feb 26;21(2):e0343772. doi: 10.1371/journal.pone.0343772 (PMC12944770; doi:10.1371/journal.pone.0343772)
Supplement: S3 Table — (DOCX) [file pone.0343772.s009.docx]

S3 Table. Summary statistics of child health and nutrition factors

| - Descriptive statistics are stratified by child stunting status (not-stunted N=438; stunted N=163) - N: total number of non‑missing observations; Values are n (%) for categorical variables (percent of non-missing observations, across both strata); Continuous variables are summarised as Median (IQR) and Mean (SD) with observed range.   - IQR: Interquartile range, SD: standard deviation - ^1^Pearson’s Chi-squared tests or Fisher’s exact test; Wilcoxon rank‑sum (continuous). Statistical significance was evaluated at α = 0.05. | | | | |
| --- | --- | --- | --- | --- |
| **CHILD HEALTH AND NUTRITION FACTORS** | ***N*** | ***Stunting status*** | | ***p-value****^1^* |
|  |  | **Not-stunted**, *n (%)* | **Stunted**, *n (%)* |  |
| Wasting status | 601 |  |  | 0.4 |
| Not-wasted |  | 427 (97.49%) | 157 (96.32%) |  |
| Wasted |  | 11 (2.511%) | 6 (3.681%) |  |
| Underweight status | 601 |  |  | <0.001 |
| Normal/Above normal weight |  | 429 (97.95%) | 131 (80.37%) |  |
| Underweight |  | 9 (2.055%) | 32 (19.63%) |  |
| Child age | 601 |  |  | <0.001 |
| Median (IQR) |  | 15 (8 - 24) | 23 (17 - 29) |  |
| Mean (SD) |  | 16 (10) | 22 (8) |  |
| Range |  | 1 - 36 | 2 - 36 |  |
| Child age group | 601 |  |  | <0.001 |
| 1 to 12 months |  | 194 (44.29%) | 21 (12.88%) |  |
| 13 to 24 months |  | 140 (31.96%) | 73 (44.79%) |  |
| 25 to 36 months |  | 104 (23.74%) | 69 (42.33%) |  |
| Child sex | 601 |  |  | <0.001 |
| Female |  | 246 (56.16%) | 65 (39.88%) |  |
| Male |  | 192 (43.84%) | 98 (60.12%) |  |
| Birth weight | 594 |  |  | <0.001 |
| Median (IQR) |  | 3200 (3000 - 3500) | 3000 (2800 - 3400) |  |
| Mean (SD) |  | 3246 (484) | 3049 (526) |  |
| Range |  | 1330 - 5000 | 1500 - 4500 |  |
| Missing |  | 1 | 6 |  |
| Child currently ill | 601 |  |  | 0.074 |
| No |  | 330 (75.34%) | 111 (68.10%) |  |
| Yes |  | 108 (24.66%) | 52 (31.90%) |  |
| Child had diarrhoea | 596 |  |  | 0.071 |
| No |  | 339 (77.93%) | 114 (70.81%) |  |
| Yes |  | 96 (22.07%) | 47 (29.19%) |  |
| Missing |  | 3 | 2 |  |
| Chronic diseases | 590 |  |  | 0.3 |
| Allergy or Asthma |  | 1 (0.233%) | 2 (1.242%) |  |
| Congenital disease |  | 5 (1.166%) | 3 (1.863%) |  |
| No chronic disease |  | 422 (98.37%) | 156 (96.89%) |  |
| Neurological illness |  | 1 (0.233%) | 0 (0%) |  |
| Missing |  | 9 | 2 |  |
| Distance to health centre | 601 |  |  | 0.021 |
| Median (IQR) |  | 2594 (1542 - 3821) | 3065 (1809 - 4257) |  |
| Mean (SD) |  | 2768 (1523) | 3129 (1644) |  |
| Range |  | 153 - 7799 | 282 - 7672 |  |
| Still Breastfeeding | 594 |  |  | <0.001 |
| No |  | 87 (20.05%) | 55 (34.38%) |  |
| Yes |  | 347 (79.95%) | 105 (65.63%) |  |
| Missing |  | 4 | 3 |  |
| Consumed types of food groups | 595 |  |  | 0.007 |
| 1 to 2 types of foods |  | 127 (29.26%) | 30 (18.63%) |  |
| 3 to 4 types of foods |  | 212 (48.85%) | 101 (62.73%) |  |
| 5 to 8 types of foods |  | 95 (21.89%) | 30 (18.63%) |  |
| Missing |  | 4 | 2 |  |
