# Supplementary material for: Spatial heterogeneity and spatially varying determinants of childhood stunting in Northern Rwanda: A cross-sectional study to inform targeted interventions
Source: PLoS One. 2026 Feb 26;21(2):e0343772. doi: 10.1371/journal.pone.0343772 (PMC12944770; doi:10.1371/journal.pone.0343772)
Supplement: S4 Table — (DOCX) [file pone.0343772.s010.docx]

S4 Table. Summary statistics of the childcare practice factors

| - Descriptive statistics are stratified by child stunting status (not-stunted N=438; stunted N=163) - N: total number of non‑missing observations; Values are n (%) for categorical variables (percent of non-missing observations, across both strata); Continuous variables are summarised as Median (IQR) and Mean (SD) with observed range.   - IQR: Interquartile range, SD: standard deviation - ^1^Pearson’s Chi-squared tests or Fisher’s exact test; Wilcoxon rank‑sum (continuous). Statistical significance was evaluated at α = 0.05. | | | | |
| --- | --- | --- | --- | --- |
| **CHILDCARE FACTORS** | ***N*** | ***Stunting status*** | | ***p-value****^1^* |
|  |  | **Not-stunted**, *n (%)* | **Stunted**, *n (%)* |  |
| Days left with another child | 600 |  |  | 0.6 |
| Median (IQR) |  | 0.00 (0.00 - 1.00) | 0.00 (0.00 - 2.00) |  |
| Mean (SD) |  | 1.05 (2.34) | 1.06 (2.11) |  |
| Range |  | 0.00 - 20.00 | 0.00 - 10.00 |  |
| Missing |  | 1 | 0 |  |
| Child was left alone more than 1 hour | 601 |  |  | 0.2 |
| Left alone more than 1 hour (1 to 10 times) |  | 135 (30.82%) | 60 (36.81%) |  |
| Never left alone |  | 303 (69.18%) | 103 (63.19%) |  |
| Child's meal was prepared by | 550 |  |  | 0.2 |
| Another person in household |  | 16 (4.030%) | 10 (6.536%) |  |
| Mother |  | 381 (95.97%) | 143 (93.46%) |  |
| Missing |  | 41 | 10 |  |
| Fed by their mother | 601 |  |  | 0.007 |
| Fed by only their mother |  | 254 (57.99%) | 71 (43.56%) |  |
| Fed by others 1 to 4 times |  | 132 (30.14%) | 65 (39.88%) |  |
| Fed by others more than 5 times |  | 52 (11.87%) | 27 (16.56%) |  |
| Given vitamin A capsule | 601 |  |  | 0.001 |
| No |  | 103 (23.52%) | 19 (11.66%) |  |
| Yes |  | 335 (76.48%) | 144 (88.34%) |  |
| Received deworming tablets | 601 |  |  | <0.001 |
| No |  | 177 (40.41%) | 26 (15.95%) |  |
| Yes |  | 261 (59.59%) | 137 (84.05%) |  |
| Used multiple micronutrient powder | 601 |  |  | 0.019 |
| Don't know |  | 1 (0.228%) | 3 (1.840%) |  |
| No |  | 323 (73.74%) | 106 (65.03%) |  |
| Yes |  | 114 (26.03%) | 54 (33.13%) |  |
| Child ever attend a nutrition clinic | 601 |  |  | 0.12 |
| Don't know |  | 1 (0.228%) | 1 (0.613%) |  |
| No |  | 337 (76.94%) | 113 (69.33%) |  |
| Yes |  | 100 (22.83%) | 49 (30.06%) |  |
| Is there any nutrition education program in the village? | 601 |  |  | 0.13 |
| Don't know |  | 5 (1.142%) | 6 (3.681%) |  |
| No |  | 140 (31.96%) | 48 (29.45%) |  |
| Yes |  | 293 (66.89%) | 109 (66.87%) |  |
| Child received any support in nutrition program | 601 |  |  | 0.5 |
| No |  | 219 (50.00%) | 77 (47.24%) |  |
| Yes |  | 219 (50.00%) | 86 (52.76%) |  |
