# Supplementary material for: Spatial heterogeneity and spatially varying determinants of childhood stunting in Northern Rwanda: A cross-sectional study to inform targeted interventions
Source: PLoS One. 2026 Feb 26;21(2):e0343772. doi: 10.1371/journal.pone.0343772 (PMC12944770; doi:10.1371/journal.pone.0343772)
Supplement: S5 Table — (DOCX) [file pone.0343772.s011.docx]

S5 Table. Summary statistics of violence against children factors

| - Descriptive statistics are stratified by child stunting status (not-stunted N=438; stunted N=163) - N: total number of non‑missing observations; Values are n (%) for categorical variables (percent of non-missing observations, across both strata); - ^1^Pearson’s Chi-squared tests or Fisher’s exact test. Statistical significance was evaluated at α = 0.05. | | | | |
| --- | --- | --- | --- | --- |
| **CHILD VIOLENCE FACTORS** | ***N*** | ***Stunting status*** | | ***p-value****^1^* |
|  |  | **Not-stunted**, *n (%)* | **Stunted**, *n (%)* |  |
| Took away privileges, forbade something he/she liked, or did not allow him/her to leave the house | 587 |  |  | 0.2 |
| Don’t know |  | 1 (0.234%) | 1 (0.629%) |  |
| No |  | 305 (71.26%) | 103 (64.78%) |  |
| Yes |  | 122 (28.50%) | 55 (34.59%) |  |
| Missing |  | 10 | 4 |  |
| Took away privileges, forbade something or did not allow other children in the family to leave the house | 534 |  |  | 0.5 |
| No |  | 227 (58.21%) | 88 (61.11%) |  |
| Yes |  | 163 (41.79%) | 56 (38.89%) |  |
| Missing |  | 48 | 19 |  |
| Shook him/her | 587 |  |  | 0.14 |
| Don’t know |  | 1 (0.234%) | 1 (0.629%) |  |
| No |  | 382 (89.25%) | 133 (83.65%) |  |
| Yes |  | 45 (10.51%) | 25 (15.72%) |  |
| Missing |  | 10 | 4 |  |
| Shouted, yelled at or screamed at the child | 587 |  |  | 0.005 |
| Don’t know |  | 1 (0.234%) | 0 (0%) |  |
| No |  | 315 (73.60%) | 97 (61.01%) |  |
| Yes |  | 112 (26.17%) | 62 (38.99%) |  |
| Missing |  | 10 | 4 |  |
| When the child did something wrong, did you or any other adult in the household give him/her something else to do | 587 |  |  | 0.040 |
| Don’t know |  | 1 (0.234%) | 1 (0.629%) |  |
| No |  | 418 (97.66%) | 149 (93.71%) |  |
| Yes |  | 9 (2.103%) | 9 (5.660%) |  |
| Missing |  | 10 | 4 |  |
| Hit him/her on the bottom or elsewhere on the body with something like a belt, hairbrush, stick or other hard object | 587 |  |  | 0.030 |
| Don’t know |  | 2 (0.467%) | 0 (0%) |  |
| No |  | 370 (86.45%) | 125 (78.62%) |  |
| Yes |  | 56 (13.08%) | 34 (21.38%) |  |
| Missing |  | 10 | 4 |  |
| Called him/her dumb, lazy or another name like that | 587 |  |  | 0.057 |
| Don’t know |  | 2 (0.467%) | 1 (0.629%) |  |
| No |  | 370 (86.45%) | 125 (78.62%) |  |
| Yes |  | 56 (13.08%) | 33 (20.75%) |  |
| Missing |  | 10 | 4 |  |
| Beat him/her up, that is hit him/her over and over as hard as one could | 587 |  |  | 0.023 |
| Don’t know |  | 4 (0.935%) | 6 (3.774%) |  |
| No |  | 419 (97.90%) | 149 (93.71%) |  |
| Yes |  | 5 (1.168%) | 4 (2.516%) |  |
| Missing |  | 10 | 4 |  |
| Beat other children in the family, that is hit him/her over and over as hard as one could | 529 |  |  | 0.034 |
| Don’t know |  | 0 (0%) | 2 (1.418%) |  |
| No |  | 366 (94.33%) | 127 (90.07%) |  |
| Yes |  | 22 (5.670%) | 12 (8.511%) |  |
| Missing |  | 50 | 22 |  |
