# Supplementary material for: Spatial heterogeneity and spatially varying determinants of childhood stunting in Northern Rwanda: A cross-sectional study to inform targeted interventions
Source: PLoS One. 2026 Feb 26;21(2):e0343772. doi: 10.1371/journal.pone.0343772 (PMC12944770; doi:10.1371/journal.pone.0343772)
Supplement: S6 Table — (DOCX) [file pone.0343772.s012.docx]

S6 Table. Summary statistics of maternal characteristics

| - Descriptive statistics are stratified by child stunting status (not-stunted N=438; stunted N=163) - N: total number of non‑missing observations; Values are n (%) for categorical variables (percent of non-missing observations, across both strata); - ^1^Pearson’s Chi-squared tests or Fisher’s exact test. Statistical significance was evaluated at α = 0.05. | | | | |
| --- | --- | --- | --- | --- |
| **MOTHER’S CHARACTERISTICS** | **N** | ***Children stunting status*** | | ***p-value****^1^* |
|  |  | **Not-stunted**, *n (%)* | **Stunted**, *n (%)* |  |
| Mother's age group | 601 |  |  | 0.8 |
| 27 - 37 |  | 225 (51.37%) | 79 (48.47%) |  |
| 17 - 26 |  | 119 (27.17%) | 45 (27.61%) |  |
| 38 and above |  | 94 (21.46%) | 39 (23.93%) |  |
| Mother's BMI | 593 |  |  | 0.084 |
| Overweight |  | 236 (54.63%) | 72 (44.72%) |  |
| Normal weight |  | 179 (41.44%) | 83 (51.55%) |  |
| Underweight |  | 17 (3.935%) | 6 (3.727%) |  |
| Missing |  | 6 | 2 |  |
| Marital status | 601 |  |  | 0.072 |
| Married |  | 312 (71.23%) | 105 (64.42%) |  |
| Cohabitating with a man/woman |  | 81 (18.49%) | 33 (20.25%) |  |
| Single |  | 39 (8.904%) | 17 (10.43%) |  |
| Divorced/Separated |  | 5 (1.142%) | 5 (3.067%) |  |
| Widowed |  | 1 (0.228%) | 3 (1.840%) |  |
| Number of miscarriages | 598 |  |  | 0.4 |
| No miscarriage |  | 383 (87.64%) | 137 (85.09%) |  |
| 1 to 4 miscarriages |  | 54 (12.36%) | 24 (14.91%) |  |
| Missing |  | 1 | 2 |  |
| Number of antenatal care visits | 600 |  |  | 0.4 |
| 4 to 6 antenatal care visits |  | 314 (71.69%) | 107 (66.05%) |  |
| 2 to 3 antenatal care visits |  | 117 (26.71%) | 52 (32.10%) |  |
| 0 to 1 antenatal care visit |  | 7 (1.598%) | 3 (1.852%) |  |
| Missing |  | 0 | 1 |  |
| Mode of delivery | 597 |  |  | 0.2 |
| Vaginally (normal) |  | 358 (82.11%) | 123 (76.40%) |  |
| Emergency caesarean section |  | 54 (12.39%) | 24 (14.91%) |  |
| Elective caesarean section |  | 12 (2.752%) | 9 (5.590%) |  |
| Vaginally with forceps |  | 10 (2.294%) | 3 (1.863%) |  |
| Vaginally with vacuum-extraction |  | 2 (0.459%) | 2 (1.242%) |  |
| Missing |  | 2 | 2 |  |
| Mother's relationship with household head | 601 |  |  | 0.3 |
| Wife |  | 390 (89.04%) | 138 (84.66%) |  |
| Daughter |  | 32 (7.306%) | 14 (8.589%) |  |
| I am head of household |  | 11 (2.511%) | 8 (4.908%) |  |
| Other family relation |  | 5 (1.142%) | 3 (1.840%) |  |
| Mother's education level | 601 |  |  | 0.073 |
| Below primary level |  | 168 (38.36%) | 51 (31.29%) |  |
| Primary level |  | 143 (32.65%) | 50 (30.67%) |  |
| Secondary level (not completed) |  | 58 (13.24%) | 31 (19.02%) |  |
| No education |  | 36 (8.219%) | 22 (13.50%) |  |
| Secondary level or higher |  | 33 (7.534%) | 9 (5.521%) |  |
| Mother's main daily activity | 600 |  |  | 0.4 |
| Farmer |  | 299 (68.26%) | 118 (72.84%) |  |
| Housewife |  | 99 (22.60%) | 34 (20.99%) |  |
| Paid worker |  | 40 (9.132%) | 10 (6.173%) |  |
| Missing |  | 0 | 1 |  |
| Have a friend that can help while ill | 594 |  |  | 0.007 |
| Always |  | 242 (56.02%) | 77 (47.53%) |  |
| Sometimes |  | 126 (29.17%) | 43 (26.54%) |  |
| Never |  | 64 (14.81%) | 42 (25.93%) |  |
| Missing |  | 6 | 1 |  |
| Have a friend that can share food | 600 |  |  | 0.12 |
| Sometimes |  | 164 (37.53%) | 63 (38.65%) |  |
| Never |  | 145 (33.18%) | 65 (39.88%) |  |
| Always |  | 128 (29.29%) | 35 (21.47%) |  |
| Missing |  | 1 | 0 |  |
| Have a friend that can share their house | 599 |  |  | 0.043 |
| Never |  | 172 (39.36%) | 77 (47.53%) |  |
| Always |  | 142 (32.49%) | 36 (22.22%) |  |
| Sometimes |  | 123 (28.15%) | 49 (30.25%) |  |
| Missing |  | 1 | 1 |  |
| Have a friend that can lend money | 600 |  |  | 0.053 |
| Never |  | 188 (43.02%) | 84 (51.53%) |  |
| Sometimes |  | 179 (40.96%) | 64 (39.26%) |  |
| Always |  | 70 (16.02%) | 15 (9.202%) |  |
| Missing |  | 1 | 0 |  |
| Have a friend that can provide help/guidance | 600 |  |  | 0.024 |
| Sometimes |  | 190 (43.48%) | 65 (39.88%) |  |
| Always |  | 190 (43.48%) | 62 (38.04%) |  |
| Never |  | 57 (13.04%) | 36 (22.09%) |  |
| Missing |  | 1 | 0 |  |
