# Supplementary material for: Spatial heterogeneity and spatially varying determinants of childhood stunting in Northern Rwanda: A cross-sectional study to inform targeted interventions
Source: PLoS One. 2026 Feb 26;21(2):e0343772. doi: 10.1371/journal.pone.0343772 (PMC12944770; doi:10.1371/journal.pone.0343772)
Supplement: S7 Table — (DOCX) [file pone.0343772.s013.docx]

S7 Table. Summary statistics of dairy production and animal health factors

| - Descriptive statistics are stratified by child stunting status (not-stunted N=438; stunted N=163) - N: total number of non‑missing observations; Values are n (%) for categorical variables (percent of non-missing observations, across both strata); - ^1^Pearson’s Chi-squared tests or Fisher’s exact test. Statistical significance was evaluated at α = 0.05. | | | | |
| --- | --- | --- | --- | --- |
| **MILK PRODUCTION AND ANIMAL HUSBANDRY FACTORS** | **N** | **Stunting status** | | **p-value***^1^* |
|  |  | **Not-stunted**, n (%) | **Stunted**, n (%) |  |
| Farming system | 155 |  |  | 0.3 |
| Intensive system |  | 105 (86.78%) | 26 (76.47%) |  |
| Semi-intensive system |  | 11 (9.091%) | 5 (14.71%) |  |
| Extensive system |  | 5 (4.132%) | 3 (8.824%) |  |
| Missing |  | 2 | 0 |  |
| Reproduction technique | 154 |  |  | 0.079 |
| Natural mating (Bull) |  | 92 (76.67%) | 32 (94.12%) |  |
| Artificial Insemination (AI) |  | 20 (16.67%) | 2 (5.882%) |  |
| Both |  | 8 (6.667%) | 0 (0%) |  |
| Missing |  | 3 | 0 |  |
| Animal house floor | 153 |  |  | 0.7 |
| Soil |  | 107 (88.43%) | 30 (93.75%) |  |
| Concrete |  | 9 (7.438%) | 1 (3.125%) |  |
| no animal house |  | 2 (1.653%) | 1 (3.125%) |  |
| wooden |  | 3 (2.479%) | 0 (0%) |  |
| Missing |  | 2 | 2 |  |
| Animal house cleaning period | 133 |  |  | 0.5 |
| Once a week |  | 35 (33.33%) | 8 (28.57%) |  |
| When needed |  | 29 (27.62%) | 12 (42.86%) |  |
| Twice a week |  | 24 (22.86%) | 4 (14.29%) |  |
| Once a day |  | 17 (16.19%) | 4 (14.29%) |  |
| Missing |  | 18 | 6 |  |
| Milking time | 156 |  |  | 0.005 |
| Once |  | 77 (63.11%) | 30 (88.24%) |  |
| Twice |  | 45 (36.89%) | 4 (11.76%) |  |
| Missing |  | 1 | 0 |  |
| Milk yield per day | 156 |  |  | >0.9 |
| <10 Litres |  | 119 (97.54%) | 33 (97.06%) |  |
| 10-20 Litres |  | 3 (2.459%) | 1 (2.941%) |  |
| Missing |  | 1 | 0 |  |
| Milking place | 156 |  |  | 0.083 |
| Cow shed |  | 110 (90.16%) | 26 (76.47%) |  |
| Open space |  | 8 (6.557%) | 6 (17.65%) |  |
| Milking parlour |  | 4 (3.279%) | 2 (5.882%) |  |
| Missing |  | 1 | 0 |  |
| Milk production purpose | 156 |  |  | 0.5 |
| Family consumption; Commercial purpose |  | 74 (60.66%) | 17 (50.00%) |  |
| Family consumption |  | 47 (38.52%) | 17 (50.00%) |  |
| Commercial purpose |  | 1 (0.820%) | 0 (0%) |  |
| Missing |  | 1 | 0 |  |
| Milking containers | 156 |  |  | >0.9 |
| Plastic buckets |  | 117 (95.90%) | 34 (100.00%) |  |
| wooden cans |  | 3 (2.459%) | 0 (0%) |  |
| Stainless steel material |  | 2 (1.639%) | 0 (0%) |  |
| Missing |  | 1 | 0 |  |
| Delivery time | 91 |  |  | 0.3 |
| Less than an hour |  | 71 (94.67%) | 14 (87.50%) |  |
| 1-2 hours |  | 4 (5.333%) | 2 (12.50%) |  |
| Missing |  | 48 | 18 |  |
| Container type for transportation time | 92 |  |  | 0.5 |
| Plastic containers |  | 64 (85.33%) | 16 (94.12%) |  |
| Stainless cans |  | 11 (14.67%) | 1 (5.882%) |  |
| Missing |  | 48 | 17 |  |
| Know how animal disease is transmitted | 157 |  |  | 0.6 |
| No |  | 64 (52.03%) | 16 (47.06%) |  |
| Yes |  | 59 (47.97%) | 18 (52.94%) |  |
| Veterinary services source | 157 |  |  | 0.2 |
| Private veterinarians |  | 82 (66.67%) | 18 (52.94%) |  |
| Sector Animal Resources Officer |  | 37 (30.08%) | 13 (38.24%) |  |
| I treat my animals by myself |  | 3 (2.439%) | 3 (8.824%) |  |
| I do not treat sick animals |  | 1 (0.813%) | 0 (0%) |  |
