# Supplementary material for: Spatial heterogeneity and spatially varying determinants of childhood stunting in Northern Rwanda: A cross-sectional study to inform targeted interventions
Source: PLoS One. 2026 Feb 26;21(2):e0343772. doi: 10.1371/journal.pone.0343772 (PMC12944770; doi:10.1371/journal.pone.0343772)
Supplement: S8 Table — (DOCX) [file pone.0343772.s014.docx]

S8 Table. Summary statistics of environmental physical factors

| - Descriptive statistics are stratified by child stunting status (not-stunted N=438; stunted N=163) - N: total number of non‑missing observations; Values are n (%) for categorical variables (percent of non-missing observations, across both strata); Continuous variables are summarised as Median (IQR) and Mean (SD) with observed range.   - IQR: Interquartile range, SD: standard deviation - ^1^Pearson’s Chi-squared tests or Fisher’s exact test; Wilcoxon rank‑sum (continuous). Statistical significance was evaluated at α = 0.05. | | | | |
| --- | --- | --- | --- | --- |
| ENVIRONMENTAL PHYSICAL FACTORS | *N* | *Stunting status* | | *p-value^1^* |
|  |  | Not-stunted  *Median (IQR)*  *Mean (SD)* | Stunted  *Median (IQR)*  *Mean (SD))* |  |
| Distance to closest health facility (in meters) | 601 |  |  | 0.021 |
| Median (IQR) |  | 2594 (1542 - 3821) | 3065 (1809 - 4257) |  |
| Mean (SD) |  | 2768 (1523) | 3129 (1644) |  |
| Range |  | 153 - 7799 | 282 - 7672 |  |
| Distance to main roads (in meters) | 601 |  |  | 0.5 |
| Median (IQR) |  | 896 (394 - 1453) | 981 (444 - 1541) |  |
| Mean (SD) |  | 1034 (822) | 1087 (856) |  |
| Range |  | 3 - 4338 | 2 - 4072 |  |
| Distance to closest market (in meters) | 601 |  |  | 0.13 |
| Median (IQR) |  | 2342 (1592 - 3140) | 2502 (1625 - 3364) |  |
| Mean (SD) |  | 2409 (1168) | 2641 (1362) |  |
| Range |  | 79 - 6003 | 145 - 6097 |  |
| Elevation (in meters) | 601 |  |  | 0.10 |
| Median (IQR) |  | 1918 (1799 - 2109) | 1971 (1814 - 2154) |  |
| Mean (SD) |  | 1946 (228) | 1984 (242) |  |
| Range |  | 1455 - 2518 | 1461 - 2539 |  |
| Mean annual rainfall (mm) | 601 |  |  | 0.5 |
| Median (IQR) |  | 1241 (1195 - 1294) | 1242 (1199 - 1303) |  |
| Mean (SD) |  | 1252 (121) | 1259 (130) |  |
| Range |  | 769 - 1743 | 745 - 1733 |  |
| Mean annual temperature (mm) | 601 |  |  | 0.080 |
| Median (IQR) |  | 17.91 (16.88 - 18.55) | 17.68 (16.76 - 18.39) |  |
| Mean (SD) |  | 17.67 (1.29) | 17.46 (1.23) |  |
| Range |  | 13.73 - 20.77 | 13.89 - 20.11 |  |
