# Supplementary material for: Spatial heterogeneity and spatially varying determinants of childhood stunting in Northern Rwanda: A cross-sectional study to inform targeted interventions
Source: PLoS One. 2026 Feb 26;21(2):e0343772. doi: 10.1371/journal.pone.0343772 (PMC12944770; doi:10.1371/journal.pone.0343772)
Supplement: S9 Table — (DOCX) [file pone.0343772.s015.docx]

S9 Table. Summary statistics for selected predictor-variables from univariable logistic regression model (odd ratios ranked by AIC, and discussed in Results Section)

| - *OR: odds ratio; 95% CI (lower) and 95% CI (upper): the lower and upper bounds of the 95% confidence interval for the OR.* - *p-value: statistical significance assessed at α = 0.05.* - *AIC: Akaike information criterion; Lower values indicate better relative model fit among the univariable models reported.* | | | | | | |
| --- | --- | --- | --- | --- | --- | --- |
| **Predictors** | **OR** | **95% CI (lower)** | **95% CI (upper)** | **p-value** | **Pseudo R^2^** | **AIC** |
| **Household socio-demographic factors** | | | | | | |
| Handwashing facility (yes) | 0.229 | 0.089 | 0.588 | 0.002 | 0.024 | 551 |
| Washes hand before preparing food (rarely) | 2.913 | 1.381 | 6.145 | 0.005 | 0.014 | 557 |
| Kind of toilet (non-improved) | 1.686 | 1.112 | 2.556 | 0.014 | 0.011 | 559 |
| Washed with soap the last 24h (yes) | 0.506 | 0.297 | 0.864 | 0.013 | 0.011 | 559 |
| Sex of household head (male) | 0.583 | 0.290 | 1.170 | 0.129 | 0.004 | 563 |
| **Household economic predictors** | | | | | | |
| Household access to electricity (yes) | 0.512 | 0.331 | 0.792 | 0.003 | 0.017 | 555 |
| Household Food Insecurity (HFIAS) | 1.351 | 1.104 | 1.652 | 0.004 | 0.015 | 556 |
| Have home garden (yes) | 0.609 | 0.392 | 0.948 | 0.028 | 0.008 | 560 |
| No milk consumption | 1.665 | 1.097 | 2.529 | 0.017 | 0.010 | 559 |
| House type (with hard materials) | 0.699 | 0.454 | 1.078 | 0.105 | 0.005 | 562 |
| **Child health and nutrition factors** | | | | | | |
| Child age | 1.962 | 1.574 | 2.446 | 0.000 | 0.070 | 525 |
| Child sex (male) | 1.900 | 1.259 | 2.866 | 0.002 | 0.017 | 555 |
| Underweight status (yes) | 8.708 | 3.768 | 20.124 | 0.000 | 0.053 | 535 |
| Birthweight | 0.654 | 0.530 | 0.807 | 0.000 | 0.029 | 548 |
| Distance to closest health facility | 1.324 | 1.083 | 1.619 | 0.006 | 0.014 | 557 |
| Had diarrhoea in last 2 weeks (yes) | 1.623 | 1.026 | 2.569 | 0.039 | 0.007 | 561 |
| Types of food consumed | 1.221 | 0.995 | 1.497 | 0.055 | 0.007 | 561 |
| **Childcare practices and presence of violence against children** | | | | | | |
| Received deworming tablets (yes) | 4.077 | 2.420 | 6.868 | 0.000 | 0.060 | 531 |
| Given vitamin A capsule(yes) | 3.203 | 1.686 | 6.086 | 0.000 | 0.028 | 549 |
| Child shouted at (yes) | 2.049 | 1.340 | 3.135 | 0.001 | 0.019 | 554 |
| Child called names (yes) | 2.200 | 1.308 | 3.700 | 0.003 | 0.015 | 556 |
| Child hit (yes) | 1.781 | 1.178 | 2.693 | 0.006 | 0.013 | 557 |
| Times child fed by others | 1.296 | 1.072 | 1.566 | 0.007 | 0.013 | 558 |
| Days left alone > 1 hour | 1.029 | 0.844 | 1.256 | 0.775 | 0.000 | 565 |
| **Maternal health and presence of violence against mothers** | | | | | | |
| Height of mother | 0.843 | 0.694 | 1.025 | 0.086 | 0.005 | 562 |
| Number of ANC visits | 0.838 | 0.690 | 1.017 | 0.074 | 0.006 | 562 |
| Number of miscarriages | 1.236 | 1.027 | 1.487 | 0.025 | 0.009 | 560 |
| Maternal social support (no help when ill) | 2.030 | 1.244 | 3.312 | 0.005 | 0.014 | 557 |
| Maternal social support (no personal support) | 1.563 | 0.985 | 2.480 | 0.058 | 0.006 | 561 |
| Decision autonomy in major purchases | 2.199 | 1.109 | 4.359 | 0.024 | 0.009 | 560 |
| Decision autonomy to visit family | 1.845 | 1.068 | 3.187 | 0.028 | 0.008 | 560 |
| Decision autonomy on earned money | 2.152 | 1.066 | 4.344 | 0.032 | 0.008 | 560 |
| Decision autonomy to refuse sexual intercourse | 0.520 | 0.339 | 0.797 | 0.003 | 0.016 | 556 |
| Alcohol before pregnancy (seldom/never) | 1.396 | 0.903 | 2.159 | 0.134 | 0.004 | 562 |
| Alcohol during pregnancy (seldom/never) | 1.110 | 0.725 | 1.700 | 0.631 | 0.000 | 564 |
| **Environmental physical factors** | | | | | | |
| Distance to the closest markets | 1.267 | 1.038 | 1.547 | 0.020 | 0.010 | 559 |
| Temperature | 0.825 | 0.676 | 1.006 | 0.058 | 0.006 | 561 |
| Elevation | 1.274 | 1.041 | 1.560 | 0.019 | 0.010 | 559 |
