# Supplementary material for: Spatial heterogeneity and spatially varying determinants of childhood stunting in Northern Rwanda: A cross-sectional study to inform targeted interventions
Source: PLoS One. 2026 Feb 26;21(2):e0343772. doi: 10.1371/journal.pone.0343772 (PMC12944770; doi:10.1371/journal.pone.0343772)
Supplement: S10 Table — (DOCX) [file pone.0343772.s016.docx]

S10 Table. Sensitivity analysis: mixed-effects logistic regression with village random intercept for predictors of childhood stunting. Results are presented as adjusted odds ratios with 95% intervals (2.5^th^ –97.5^th^ percentiles).

| - *aOR: Adjusted odds ratio; 95% CI (lower) and 95% CI (upper): Lower and upper bounds of the 95% confidence interval, defined by the 2.5^th^ and 97.5^th^ percentiles of the posterior distribution.* - *Posterior mean (log-odds): Estimated fixed-effect coefficient on the log-odds scale.* - *Posterior SD: Posterior standard deviation of the log-odds coefficient.* | | | | | |
| --- | --- | --- | --- | --- | --- |
| **Predictor** | **aOR** | **95% CI (lower)** | **95% CI (upper)** | **Posterior mean (log-odds)** | **Posterior SD** |
| Intercept | 0.093 | 0.074 | 0.12 | -2.380 | 0.116 |
| Child age | 2.61 | 2.08 | 3.28 | 0.960 | 0.117 |
| Underweight status | 15.41 | 6.41 | 37.01 | 2.735 | 0.447 |
| Child sex (Male) | 2.79 | 2.04 | 3.80 | 1.025 | 0.158 |
| Birthweight | 0.75 | 0.59 | 0.95 | -0.288 | 0.120 |
| Days cared for by another child | 0.76 | 0.61 | 0.96 | -0.271 | 0.115 |
| Days left alone >1 hour | 0.75 | 0.60 | 0.94 | -0.285 | 0.114 |
| Reading to the child (yes) | 1.52 | 1.07 | 2.18 | 0.421 | 0.182 |
| Times child fed by others | 1.11 | 0.89 | 1.38 | 0.103 | 0.112 |
| Shaking children | 0.61 | 0.37 | 1.02 | -0.488 | 0.266 |
| Types of food consumed | 1.32 | 1.02 | 1.69 | 0.275 | 0.128 |
| Milk consumption (none) | 1.51 | 1.13 | 2.03 | 0.415 | 0.149 |
| Distance to nearest health centre (m) | 1.28 | 1.03 | 1.59 | 0.248 | 0.111 |
| Elevation (m) | 1.31 | 1.04 | 1.64 | 0.266 | 0.117 |
| Handwashing facility (yes) | 0.30 | 0.12 | 0.72 | -1.213 | 0.451 |
| Household electricity access (yes) | 0.39 | 0.26 | 0.60 | -0.934 | 0.214 |
| Household Food Insecurity (HFIAS) | 1.23 | 0.99 | 1.54 | 0.209 | 0.113 |
| Education level (secondary, not complete) | 1.76 | 0.96 | 3.24 | 0.567 | 0.311 |
| Number of prior miscarriages | 1.24 | 1.00 | 1.55 | 0.219 | 0.114 |
| Number of ANC visits | 0.90 | 0.71 | 1.13 | -0.110 | 0.120 |
| Number born by caesarean | 1.18 | 0.95 | 1.48 | 0.169 | 0.113 |
| Ability to refuse sexual intercourse (yes) | 0.64 | 0.49 | 0.85 | -0.443 | 0.141 |
| Maternal social support (sometimes has help) | 1.83 | 1.17 | 2.86 | 0.602 | 0.229 |
| Mother’s headache (yes) | 1.54 | 0.98 | 2.43 | 0.435 | 0.232 |
| Alcohol before pregnancy (seldom/never) | 1.68 | 1.27 | 2.23 | 0.521 | 0.144 |
